# Localization of Fe‐S Biosynthesis Machinery in *Cryptosporidium parvum* Mitosome

**DOI:** 10.1111/jeu.12663

**Published:** 2018-07-12

**Authors:** Christopher N. Miller, Lyne Jossé, Anastasios D. Tsaousis

**Affiliations:** ^1^ Laboratory of Molecular & Evolutionary Parasitology RAPID Group School of Biosciences University of Kent Canterbury UK

**Keywords:** Apicomplexans, immunofluorescence assay, iron–sulfur clusters, mitochondria, mitosomes

## Abstract

*Cryptosporidium* is a protozoan, apicomplexan, parasite that poses significant risk to humans and animals, as a common cause of potentially fatal diarrhea in immunodeficient hosts. The parasites have evolved a number of unique biological features that allow them to thrive in a highly specialized parasitic lifestyle. For example, the genome of *Cryptosporidium parvum* is highly reduced, encoding only 3,805 proteins, which is also reflected in its reduced cellular and organellar content and functions. As such, its remnant mitochondrion, dubbed a mitosome, is one of the smallest mitochondria yet found. While numerous studies have attempted to discover the function(s) of the *C. parvum* mitosome, most of them have been focused on in silico predictions. Here, we have localized components of a biochemical pathway in the *C. parvum* mitosome, in our investigations into the functions of this peculiar mitochondrial organelle. We have shown that three proteins involved in the mitochondrial iron‐sulfur cluster biosynthetic pathway are localized in the organelle, and one of them can functionally replace its yeast homolog. Thus, it seems that the *C. parvum* mitosome is involved in iron‐sulfur cluster biosynthesis, supporting the organellar and cytosolic apoproteins. These results spearhead further research on elucidating the functions of the mitosome and broaden our understanding in the minimalistic adaptations of these organelles.

IRON (Fe) and sulfur (S) are vital elements for every living cell. While iron‐sulfur clusters can be spontaneously formed in nature, both elements, when unbound, are toxic to the living cell, and thus specialized machineries have developed for their assembly. Current studies have identified four different biosynthetic pathways responsible for the assembly of iron‐sulfur clusters. In eukaryotes, the most common biosynthetic pathways are the Iron Sulfur Cluster (ISC) machinery that is present in most mitochondria investigated so far (Lill et al. 2012) in addition to the Cytosolic Iron‐sulfur cluster Assembly (CIA) machinery, components of which are found in the cytosol of all studied eukaryotes (Tsaousis et al. 2014). Furthermore, the SUlFur mobilization (SUF) machinery is localized in the plastids of plastid bearing organisms (including those apicomplexans, which still maintain their apicoplast), but it has recently been found to be present in various protists as well, including *Blastocystis* (cytosolic localization; Tsaousis et al. 2012)*, Pygsuia* (cytosolic/mitochondrial localization; Stairs et al. 2014) and *Stygiella* (Leger et al. 2016). Even in the only currently confirmed amitochondriate organism, *Monocercomonoides*, a diverse homolog of the SUF machinery has been identified (Karnkowska et al. 2016). Lastly, components of the nitrogen fixation (NIF) machinery are encoded by the genomes of *Entamoeba* (Ali et al. 2004; van der Giezen et al. 2004) and its free‐living relative *Mastigamoeba* (Nyvltova et al. 2013).

The common feature among these machineries is the existence of three major components: a sulfur donor, an iron donor, and a scaffold protein for the assembly of the clusters. In the ISC pathway, these roles have been undertaken by the IscS, Frataxin, and IscU proteins, respectively. In eukaryotes, these proteins have been thoroughly characterized in humans (for review, see Rouault and Maio 2017), yeast (for review, see Braymer and Lill 2017), and trypanosomes (Basu et al. 2016). Recent reports have functionally characterized these proteins in other protists, including *Blastocystis* (Tsaousis et al. 2012) and microsporidia (Freibert et al. 2017; Goldberg et al. 2008).

Among apicomplexans, three of the different types of Fe‐S cluster assembly machineries can be found (Ali and Nozaki 2013): the mitochondrial ISC machinery, the apicoplast SUF machinery, and the cytosolic CIA machinery. To the best of our knowledge, the only studies on Fe‐S cluster assembly machineries in apicomplexans have been focused on the apicoplast‐localized SUF machinery in *Plasmodium* species (Charan et al. 2017). These studies have demonstrated the importance of this pathway in the survival of the parasite: the SUF machinery supports the apicoplast‐specific apoproteins (Gisselberg et al. 2013), some of which are vital for the sustainability of the parasites (Charan et al. 2014; Haussig et al. 2014). Importantly, however, with the exception of specific prediction analyses (Dellibovi‐Ragheb et al. 2013; Seeber 2002), functional investigations of the mitochondrial ISC machinery of apicomplexan parasites are completely absent.

*Cryptosporidium* is an intracellular, but extracytoplasmic apicomplexan parasite recently classified as a gregarine (Ryan et al. 2016). The parasite harbors a remnant mitochondrion, the mitosome, whose function(s) are still under debate (Mogi and Kita 2010). *Cryptosporidium parvum* and *C. hominis* mitosomes lack mitochondrial DNA, TCA cycle enzymes (with the exception of malate dehydrogenase), pyruvate dehydrogenase, complex 3 and 4 of the electron transport chain, and ATP synthase subunits with the exception of α and β (Liu et al. 2016; Mogi and Kita 2010). With the exclusion of two chaperone proteins (Cpn60 and Hsp70) that have been localized to the mitosome of the *C. parvum* sporozoite stage (Riordan et al. 2003; Slapeta and Keithly 2004), studies showing localization or biochemical characterization of *Cryptosporidium* mitochondria are lacking. With regard to the Fe‐S cluster assembly machinery, the only published study demonstrated heterologous localization of the *C. parvum* sulfur donor protein IscS and the scaffold protein IscU in *Saccharomyces cerevisiae* mitochondria (LaGier et al. 2003). Thus, direct localization studies of this machinery in *Cryptosporidium* are nonexistent.

Here, we employed various tools to characterize the ISC machinery of *Cryptosporidium parvum*. First, we generated specific antibodies against *C. parvum* IscS, IscU, and Frataxin. We then used an in vitro culture that can propagate the parasite in long term (Miller et al. 2018) to localize the proteins using western blotting and indirect immunofluorescence microscopy. This study represents the first localization of ISC biosynthetic pathway components in a *Cryptosporidium* mitosome, using not only the more frequently studied sporozoite stage but also other life stages of the parasite. As a result, this work leads the way to further studies on characterizing the peculiar mitochondrion of *Cryptosporidium*.

## Materials and Methods

### Bioinformatics analyses

To identify candidate ISC pathway proteins, we used CryptoDB (Heiges et al. 2006). BLASTP (Boratyn et al. 2012) were employed to search the database and identify full‐length sequences of the protein coding genes involved in the ISC pathway. The *Cryptosporidium* amino acid sequences were then aligned with homologous sequences from *Homo sapiens, Saccharomyces cerevisiae*, and *Escherichia coli* using MUSCLE (Edgar 2004). The secondary structures of the proteins were predicted using a combination of PSIPRED (McGuffin et al. 2000) and HMMTOP (Tusnady and Simon 2001). For the *in silico* prediction of potential mitochondrion‐targeted proteins, we employed MitoFates (Fukasawa et al. 2015), MitoProt (Claros 1995), Predotar (Small et al. 2004), and TargetP (Emanuelsson et al. 2007).

### *Cryptosporidium parvum* culturing and infection

The human cell line COLO‐680N was cultivated in RPMI‐1640 medium supplemented with 10% FBS, 100 U/ml of penicillin, 100 μg/ml of streptomycin. *Cryptosporidium parvum* oocysts (Iowa strain) were obtained from Bunch Grass Farm (Deary, Idaho, USA). For a typical infection, 1 × 10^5^ oocysts were used to infect 25‐cm^2^ cell culture flasks at between 70% and 80% confluency (1.7 to 2 × 10^6^ cells) giving a multiplicity of infection (MOI) of approximately 0.2. When smaller cell surfaces were used, the number of COLO‐680N and oocysts were altered proportionally. Excystation was done according to Miller et al. (2018).

### Generation of antibodies used in this study

The predicted *Cryptosporidium parvum* frataxin, IscS, and IscU genes were amplified from genomic DNA (reference accession number XM_625594, AY029212, XM_627477, respectively) and cloned into an *E. coli* pET14b expression vector as a *Nde*I/*Bam*HI fragment allowing His N‐terminal tagging. The recombinant DNA constructs were transformed chemically in the *E. coli* BL21(DE3) pLysS expression strain (Novagen, Millipore (UK) Limited, Watford, UK). Protein expression was done in 2 L of LB, seeded with an overnight inoculum and left at 37 °C for 16 h or until an OD_600_ reached 0.4–0.6, at which point IPTG was added to a final concentration of 0.5 mM. The cultures were grown for a further 4 h before centrifuging at 3,000 *g* for 30 min. The supernatant was then discarded and the pellet washed twice in PBS. The pellet was then weighed, before resuspending it in Novagen BugBuster reagent (Novagen) at 5 ml/g of pellet and 1 μl/ml of Benzonase, which was added to the mixture to inhibit proteolysis. The mixture was left to shake at a low setting for 20 min at room temperature and subsequently centrifuged at 16,000 *g* for 20 min at 4 °C. The His‐tagged proteins (Frataxin, IscU and IscS) were purified on Nickel affinity column. Eluted proteins were run onto a 15% SDS‐PAGE gels and the bands corresponding to the proteins of interest were then excised from the gels and used to inoculate a rabbit (for IscS) or rats (for IscU or Frataxin), which subsequently produced polyclonal antibodies to the target proteins.

For generation of antibodies against *C. parvum* 60S ribosomal protein L23A, peptide 1 (Aa‐29–43: nh2‐C+VKNSKRSSTTKIRTR‐conh2) and peptide 2 (58–73: ac‐PKYERKSIKTQKSLDC‐conh2) were synthesized. For peptide 1, a cysteine was added to target the coupling site at the carrier protein KHL (Keyhole Limpet Hemocyanin). BLASTP search revealed that neither peptide was present in the human protein homolog. Notably, peptide 1 was 100% identical with *C. ubiquitum* and *C. hominis,* but only 48% with *C. muris*; while peptide 2 shared 100% identity with *C. hominis* and 88% with *C. ubiquitum*. The conjugated peptides were raised in laying hens and isolated from the yolk of their eggs (Eurogentec, Southampton, UK).

### Protein extraction and western blot analysis

*Cryptosporidium parvum* oocysts (5× 10^7^) and COLO‐68ON cells (4–5 × 10^5^) were lysed in 160 μl buffer L [20 mM Tris–Cl (pH 7.5), 10 mM EDTA, 10 mM EGTA, 150 mM NaCl and 1% (w/v) Triton and one tablet of protease inhibitor cocktail (Roche Products Limited, Welwyn Garden City, UK)] and sonicated at 14 watts/cm^2^, 3 × 30 s to break down the oocyst envelope, and shear the DNA. Subsequently, 40 μl of 5× SDS loading buffer (5% β‐mercaptoethanol, 0.02% bromophenol blue, 30% glycerol, 10% sodium dodecyl sulfate, 250 mM, pH 6.8) were added. Ten microliters of total proteins were analyzed on 15% SDS‐PAGE gels and stained with Coomassie Blue or transferred onto PVDF membrane (Roche Products Limited, Welwyn Garden City, UK) and probed with primary antibodies at 1:3,000 dilution. Anti‐chicken HPR‐conjugated antibodies (Sigma‐Aldrich Company Ltd, Gillingham, UK) were used as a secondary antibody (1:50,000) and the peroxidase reaction was catalyzed using ECL prime reagent (GE Healthcare, Amersham, UK).

### Immunofluorescence microscopy

COLO‐680N infection was carried out in Nunc Lab‐Tek chamber slides (Permanox, 4.2 cm^2^), Nalgene Nunc International, Rochester, NY, USA. At harvesting points, cultures were washed with 1× PBS and then fixed in methanol for 10 min at room temperature. Next, the methanol was removed and the cells were permeabilized with 0.002% Triton‐X100 in 1× PBS at room temperature for 30 min. Cells were then washed three times prior to incubation for 1 h with *Cryptosporidium*‐specific commercial antibodies Crypt‐a‐glo (Waterborne^TM^; New Orleans, LA, USA, specific to *Cryptosporidium* oocysts) in dilution 1:10, and Sporo‐glo (Waterborne^TM^; New Orleans, specific to *Cryptosporidium* intracellular life cycle stages) in dilution 1:10 (Miller et al. 2018). Cells were subsequently washed three times with 1 × PBS, prior to incubation overnight with *Cryptosporidium*‐specific antibodies (anti‐Frataxin, dilution 1:200; anti‐IscS, dilution 1:100; anti‐IscU, dilution 1: 50). Cells were washed three times with 1xPBS prior to incubation with secondary antibodies Alexa 488 (Green; Molecular Probes, Thermo Fisher Scientific, Waltham, MA, USA) or Alexa 594 (Red; Molecular Probes, Thermo Fisher Scientific) at 1:200, for one hour. Cells were washed a further three times with 1× PBS and mounted with ProLong Gold antifade reagent (Thermo‐Fischer Scientific), using high‐performance coverslips (Carl Zeiss Microscopy, Carl Zeiss AG, Oberkochen, Germany). Slides were visualized by fluorescence microscopy using a Zeiss Elyra P1 confocal microscope (Carl Zeiss AG).

### Yeast complementation studies of IscS

#### Yeast strains and growth conditions

The *S. cerevisiae* strain used in this study was the heterozygous diploid YCL017C (*MATa/MATalpha his3delta1/his3delta1 leu2delta0/leu2delta0 lys2delta0/+ met15delta0/+ ura3delta0/ura3delta0 deltaNFS1:: KAN* ^*R*^ *),* the knockout diploid strain was purchased from Dharmacon, Horizon Discovery, Cambridge, UK, from the Yeast HetDip Knock Out collection, Clone ID: 23424. Typically, the YCL017C strain was grown at 30 °C in YEPD (1% Yeast Extract, 2% Peptone, 2% dextrose or glucose) in the presence of 200 μg/ml geneticin (G418).

#### Plasmid construction

The predicted *Cryptosporidium parvum* IscS gene was amplified from genomic DNA (reference accession number AY029212) and cloned into *E. coli* pET14b expression vector. For yeast complementation experiment, the fragment was subcloned into the bi‐directional yeast expression vector pBEVY‐L (Miller et al. 1998) as a *Sma*I/ *Sac*I fragment. pBEVY‐L carries the LEU2 selection marker and can be propagated in synthetic complete media lacking leucine.

#### Yeast transformation and selection

*Saccharomyces cerevisiae* cells were transformed from overnight culture using the lithium acetate/ single stranded (ss) carrier DNA/ polyethylene glycol (PEG) method adapted from Gietz and Woods (Gietz and Woods 2002). Briefly, for each transformation, 1 ml of overnight culture was harvested at 13,000 *g* for 30 s and the pellet was washed once in sterile water. Then, reagents were added in the following order: 240 μl PEG 4000, 36 μl 1 M LiAc, 10 μl of 10 mg/ml ss DNA (recently boiled and sonicated), 2.5 μl β‐ mercaptoethanol, 2 μl plasmid (100–500 ng/μl), 70 μl sterile water. The mixture was left for 20 min at room temperature prior to heat shock at 42 °C for 20 min, then briefly spun down at 13,000 *g* to pellet the cells. Cells were resuspended in 200 μl of sterile water and 50 and 100 μl were plated out. Transformants were selected on synthetic dropout plates lacking leucine.

#### Sporulation (Adapted from Curran and Bugeja 2014)

Yeast transformants (one colony) were grown overnight in synthetic complete medium lacking leucine (Synthetic Complete (SC), ‐leu), with 2% glucose. Cells were then diluted 1/50 and 1/100 in 1 ml in SC, ‐leu medium enriched with 5% glucose and grown at 30 °C for approximately 8 h or until optical density reached OD_600 _= 0.1 to 0.3. Cells were spun at 3,000 *g* for 5 min and washed twice in sterile water. Cells were then resuspended in 1 ml of sterile 1% potassium acetate and left with vigorous shaking at 25 °C for 4–5 d. After 4 d, cells were examined under the microscope to evaluate the formation of tetrad cells or asci (in excess of 30%).

#### Isolation of spores (Adapted from Curran and Bugeja 2014)

To remove spores from asci, tetrad cells and nontetrad cells were harvested and resuspended in sterile water. Fifty microliters of lyticase (from a 100,000 units/ml stock) were added to digest the ascal wall, and the reaction was incubated at 37 °C for 10–15 min. One volume of sterile mineral oil was added and the mixture was vortexed for 2 min to mechanically disrupt asci and release spores. The oil/water mixture was spun at 3,000 *g* for 1 min in order to separate both phases. The mineral oil layer was enriched with hydrophobic spores, while the majority of vegetative cells and tetrad cells remained in the water. Spores were carefully removed from the mineral oil phase, mixed with sterile water and plated on appropriate media at dilutions ranging from 1/10 to 1/50. Spores were first plated out randomly on YEPD plates. Colonies were then individually picked up and transferred onto SC, ‐leu plates or YEPD, 200 μg/ml G418 plates. Cells (haploid or diploid) able to grow on both types of plates have a disrupted *NFS1* gene (replaced by kanamycin marker) and carry the *C. parvum* putative homolog IscS (on pBEVY‐L).

#### Discrimination between haploid and diploid yeast transformants

This method was adapted from Huxley et al. (1990) and used here to ensure that cells growing on SC‐leu and YEPD, 200 μg/ml G418 were haploids and not diploids, thus demonstrating that *C. parvum* IscS can rescue delta *NSF1* lethality in *S. cerevisiae*. For this analysis, the following primers were used: (1) 5′‐AGTCACATCAAGATCGTTTATGG‐3′, (2) 5′‐GCACGGAATATGGGACTACTTCG‐3′, (3) 5′‐ACTCCACTTCAAGTAAGAGTTTG‐3′. PCR mix: 1/5 colony, 0.2 μl each primer (100 pmole/μl), 2.0 μl dNTPs (10 mM), 0.25 μl GoTaq DNA polymerase, water to 20 μl. Hot start at 92 °C for 5 min, cycles 30 s at 92 °C, 30 s at 55 °C, 1 min at 72 °C. Expected product sizes were 404 bp for a Mat alpha haploid and 544 bp for a MAT a haploid. The presence of both bands indicated the presence of a diploid, which was not removed during the isolation of spores.

## Results

### *Cryptosporidium* ISC proteins

Due to the minimalistic properties of *Cryptosporidium parvum* mitosome, we sought to investigate the assembly of proteins constituting the ISC pathway of the parasite. Using BLASTP approach against various databases (nr and CryptoDB), we have identified a wide range of the components of the mitochondrial ISC machinery encoded by the *C. parvum* genome (Table S1). Interestingly, comparison with the *C. andersoni* and the *C. ubiquitum* genomes demonstrated that these species have a more complete range of components. To investigate the presence of compartment‐specific targeting signals, all the predicted *Cryptosporidium* spp. ISC proteins were analyzed using four different prediction programs (Table S2). These analyses revealed that all algorithms were abled to predict localization of IscS and IscU proteins in the mitochondria of all *Cryptosporidium* species, with the exception of MitoProt, and Frataxin was not predicted to be mitochondrial.

To predict the properties of these three proteins, the full‐length amino acid sequences of *Cryptosporidium* spp. IscS, IscU, and Frataxin proteins were aligned with yeast, human, and *E. coli* ISC proteins. Sequence alignment of all three proteins revealed conserved features associated with canonical ISC functions (Figs S1–S3). Interestingly, while all IscS homologs were predicted to harbor an N‐terminal mitochondrial targeting signal (MTS) (Fig. S1), in the case of the IscU, only *C. ubiquitum* was predicted to have one (Fig. S2). In the case of Frataxin, only *C. ubiquitum* and *C. andersoni* along with the yeast homolog were predicted to have an N‐terminal MTS (Fig. S3).

### *Cryptosporidium* ISC machinery is expressed

**Figure 1:**
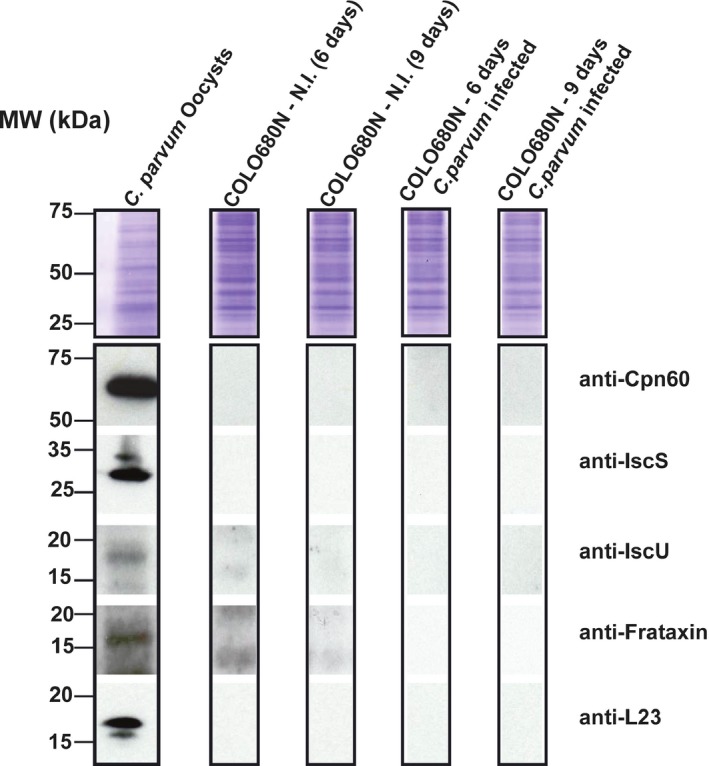
Resolution of total proteins by SDS‐PAGE and downstream analysis: Top panel, the protein gel was stained with Coomassie Blue, as a loading control reference. Other panels, Western blot analysis was done to probe for individual proteins, using corresponding antibodies against: Cpn60, IscS, IscU, frataxin, and L23A. *Cryptosporidium parvum* oocysts were isolated from calves infected with the *C. parvum* Iowa strain. COLO680N‐N.I corresponds to host control cells. COLO680N infected corresponds to host cells infected with *C. parvum*. Host cells noninfected and infected were cultured in parallel and harvested at day 6 and day 9 postinfection.

To examine the expression and localization of these proteins in *Cryptosporidium*, we generated specific antibodies against the complete *C. parvum* IscS, IscU, and Frataxin proteins (named *Cp*IscS, *Cp*IscU, and *Cp*Frataxin, respectively). To investigate the specificity of the antisera using western blots, we used proteins that were extracted from *C. parvum* Iowa strain oocysts. Western blotting experiments using the previously published anti‐*Cp*Cpn60 (Riordan et al. 2003) and newly generated anti‐*Cp*L23A (control), anti‐*Cp*IscS, anti‐*Cp*IscU, and anti‐*Cp*Frataxin antisera showed specific bands in molecular weights of 17, 29, 17, 16 kDa, respectively, in the protein extracts from the *C. parvum* oocysts, which were not present in the protein extracts from the noninfected COLO‐680N cells (Fig. 1).

### The ISC machinery is localized in *Cryptosporidium* mitosomes

To determine the cellular location of the *C. parvum* ISC proteins, indirect immunofluorescence analyses were carried out on 10‐day infected COLO‐680N cells and/or on isolated oocysts. Co‐localization of the various proteins within the oocyst of *C. parvum* was achieved by co‐staining with Crypt‐a‐glo, a FITC conjugated, monoclonal antibody with high specificity for a *Cryptosporidium* oocyst wall protein (Miller et al. 2018). Localization within other stages of *C. parvum*, sporozoites and intracellular stages, for example, was achieved by co‐staining with Sporo‐glo, a sensitive, FITC‐conjugated polyclonal antibody raised against *C. parvum* sporozoites.

### *Cp*IscS

**Figure 2:**
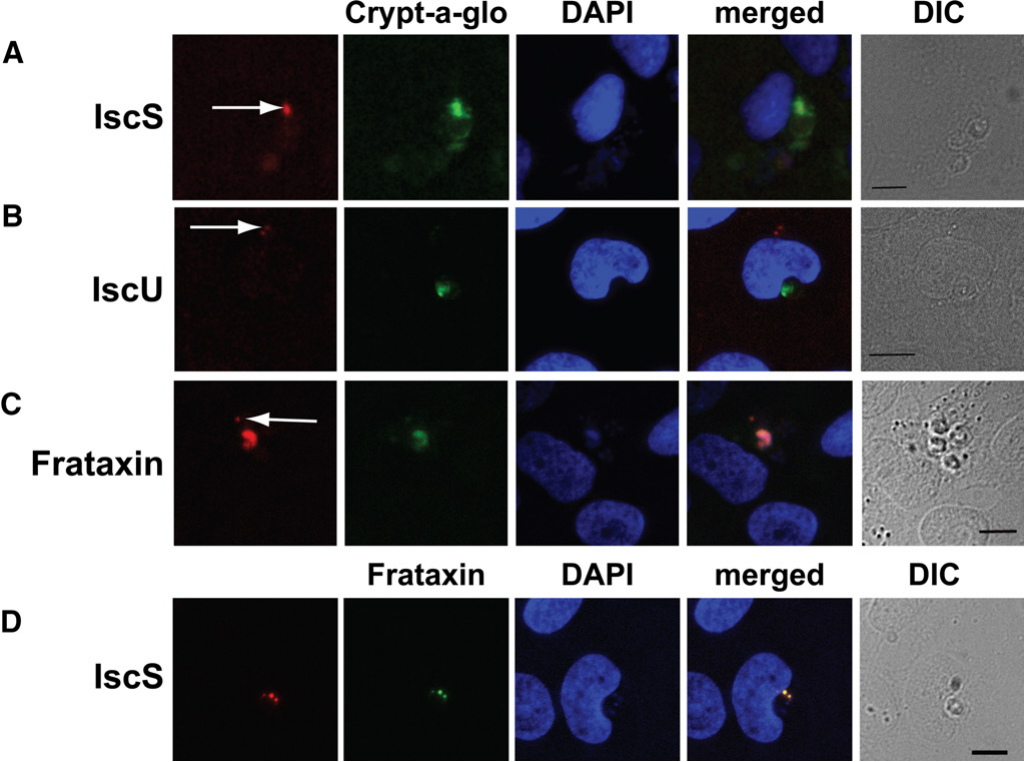
Cellular localization of IscS, IscU, and Frataxin‐like proteins in *Cryptosporidium parvum* by immunofluorescence microscopy. The nuclei of COLO‐680N host cells (large nuclei) and parasites (small nuclei) were labeled with DAPI (blue). (**A**) Rabbit antisera to *Cp*IscS detects discreet structures (red) on *C. parvum*, whereas the Crypt‐a‐glo detects (green) the parasites. (**B**) Rat antisera to CpIscU detect discreet structures (red) on *C. parvum*, whereas the Crypt‐a‐glo detects (green) the parasites. (**C**) Rat antisera to *Cp*Frataxin detect discreet structures (red) on *C. parvum*, whereas the Crypt‐a‐glo detects (green) the parasites. Differential interference contrast (DIC) images of the cells used for immunofluorescence. (**D**) Rabbit antisera to *Cp*IscS detects discreet structures (red) on *C. parvum* that colocalize with the same discreet structures detected by *Cp*Frataxin rat antisera (green). Scale bar 10 μm.

Incubation of the infected cultures with the anti‐*Cp*IscS revealed a life cycle‐dependent localization of *Cp*IscS in immunofluorescent assays (Fig. 2A; Fig. S4). During the extracellular stages (e.g. sporozoites and merozoites), the signal from IscS was punctate (Fig. S4) and restricted to regions typically between 500 and 800 nm in diameter. DAPI‐stained oocysts of *C. parvum* showed successful permeation as the four nuclei of the contained sporozoites could reliably be observed in an oocyst. However, the absence of detectable fluorescence, within these sporozoites, suggested absence of IscS signal. It may, therefore, be assumed that IscS expression levels are either negligible so as not to be detectable or IscS is not being expressed at all, while the sporozoite remains within a matured (thick) oocyst. Immunofluorescent assays of the other intracellular stages revealed that while IscS remained expressed at detectable levels, its localization did not remain consistent. In detectable extracellular life cycle stages, determined as any detectable Sporo‐Glo not within/closely associated with a host cell and <4 μm in diameter, IscS was found concentrated in a region similar to that observed in the sporozoites. This indicates the presence of an organelle matching the physical descriptions of the mitosome, corroborating the hypothesis that the ISC pathway localizes to the mitosome of *C. parvum*. However, in occasions, immunofluorescent assays of intracellular life cycle stages, determined by positive Sporo‐Glo detection, proximity to host nucleus, amorphous/round shape, and an approximate parasite size exceeding 4 μm showed that IscS was more diffuse (Fig. S4). Indeed, it was hard to discern any regularly observable structural association with IscS expression in intracellular stages. Furthermore, in areas where later developmental stages of *C. parvum* were present, IscS staining appeared to decrease inversely proportional to the diffusion of genetic material, indicating that IscS expression declined as the sporozoites were formed.

### IscU

Anti‐*Cp*IscU localization studies produced similar patterns to that of *Cp*IscS when observed via immunofluorescent assays (Fig. 2B; Fig. S5). Life‐cycle stage‐dependent expression continued to be present; with Crypt‐a‐glo stained oocysts presenting DAPI‐stained sporozoite nuclei, but no detectable *Cp*IscU signal. Conversely, several extracellular life cycle stages could be detected by residual Crypt‐a‐glo labeling or punctate DAPI stain, which did display detectable levels of *Cp*IscU. However, in occasions, *Cp*IscU signal appeared to be more diffused than that of *Cp*IscS throughout the life cycle of the parasite (Fig. S5). This could indicate that *Cp*IscU may not only be localized to a specific structure within the parasite, or at least not exclusively during the development of *C. parvum*.

### Frataxin

**Figure 3:**
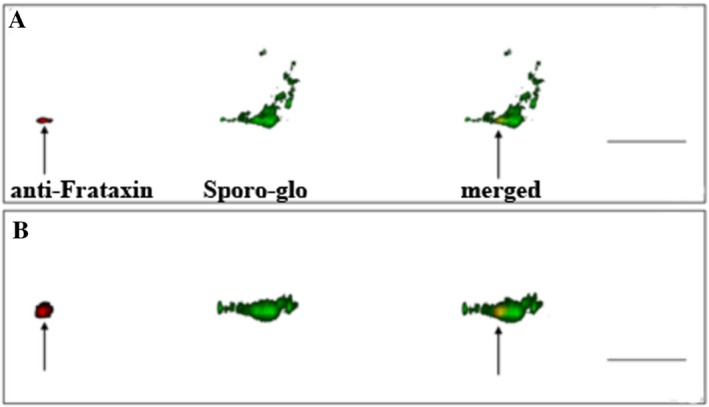
Confocal microscopy of an infected COLO‐680N culture, labeled with anti‐CpFxn (red) and SporoGlo (green). (**A**) A 3D, high‐resolution confocal image of a *Cryptosporidium parvum* extracellular life cycle stage in an infected COLO‐680N culture. Clear labeling of CpFrataxin (red) highlights an approximately 700‐nm wide oval (arrow) at the apical end of the parasite, a red outline has been drawn to illustrate the shape of the parasite and to highlight the location of the mitosome. (**B**) The same image rotated through 90° on the *x*‐axis. Scale bar: 5 μm.

Anti‐*Cp*Frataxin localization studies showed similar patterns to those of *Cp*IscS and *Cp*IscU. *Cp*Frataxin antibody labeling of infected cultures showed, again, a lack of *Cp*Frataxin in mature oocysts, but abundance in other life cycle stages (Fig. 2C; Fig. S6). Most notably, CpFrataxin signal remained intense even in late stage zygotes, denoted by the intense Crypt‐a‐glo signal that lacked the characteristic “ring” of a mature oocyst. This is in contrast to both *Cp*IscS and *Cp*IscU that showed decreasing levels of expression leading up to zygote maturation. *Cp*Frataxin signal also remained relatively diffuse throughout the life cycle, although dramatically more so in the intracellular stages, similarly to *Cp*IscU. Despite this, fluorescence microscopy using both anti‐*Cp*Frataxin the anti‐*Cp*IscS antibodies demonstrated clear co‐localization of both proteins in distinct, punctuated structures within *C. parvum* intracellular stages (Fig. 2D). In addition, using confocal microscopy, the *Cp*Frataxin labeling in sporozoites appeared considerably more localized than any other life cycle stage, appearing to congregate in a defined, approximately 500‐nm oval near the apical end of the parasite (Fig. 3; Video S1).

### *Cryptosporidium* IscS can functionally replace the yeast homolog

**Figure 4:**
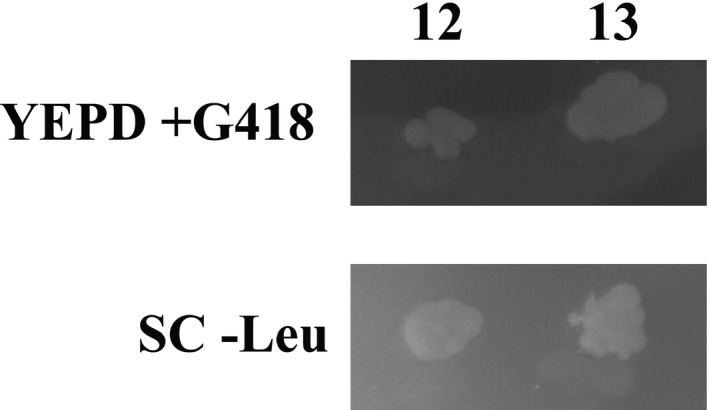
Complementation of *Saccharomyces cerevisiae nfs1:: KANMX4* knockout haploids with *Cryptosporidium parvum* IscS and random spore analysis. Top panel, spores‐derived colonies were streaked onto YEPD, 200 μg/ml G418 plates to select for clones that lack the *NFS1* yeast gene. Bottom panel, spores‐derived were streaked onto SC‐Leu plates, in parallel to select for strains that had taken in the plasmid pBEVY‐L/*C. parvum* IscS. All colonies were checked for haploidy (see Fig. S7).

The CpIscS recombinant construct was transformed into a heterozygous *Sc*Nfs1p (*Sc*IscS) knockout, strain YCL017C, where a homozygous knockout is lethal. In the knockout strain of YCL017C, the Nfs1 gene was replaced by a G418 resistance marker on chromosome III. Colonies of transformants were grown on SC‐leucine plates to select for *S. cerevisiae* containing the plasmid pBEVY‐L/IscS. Sporulation to isolate haploid, which might rescue Nfs1 lethality, in the presence of *C. parvum* IscS, was carried out in a nitrogen‐deprived liquid culture. Spores were removed from the ascus by enzymatic digestion of the cell wall, separated from nondigested tetrads and plated out on YEPD plates, as described in Materials and Methods. Spores were first plated out on YEPD before being individually picked onto SC‐leu and YEPD/G418 and tested for mating type via colony PCR (Fig. S7). We observed that *Cp*IscS recovered the fatal *Sc*Nfs1 knockout, as determined from the matching colonies present on both leucine deficient media and G418 resistance plates (Fig. 4).

## Discussion

The recently established system for maintaining *C. parvum* in culture allows investigating further the cell biology of this parasite, within and outside the host (Miller et al. 2018). During invasion, *Cryptosporidium* becomes intracellular, but extracytoplasmic, and it has a constant dependence on the host. Due to this reliance, the parasite has reduced metabolic capabilities, including mitochondrial functions, as shown by the absence of most canonical pathways. For example, *C. parvum* and *C. hominis* remnant mitochondria (mitosomes) have lost the protein coding genes involved in the TCA cycle, thus ATP synthesis depends solely on glycolysis (Abrahamsen et al. 2004; Xu et al. 2004). The only complete biosynthetic pathways predicted to be present in these mitosomes are those of ubiquinone and Fe‐S cluster biosynthesis. A more extreme case is the *C. ubiquitum* mitosome, which also lacks the capacity of ubiquinone biosynthesis (Liu et al. 2016).

Here, we sought to investigate the localization and function of proteins involved in the mitochondrial Fe‐S cluster (ISC) biosynthesis in *C. parvum*. We amplified, cloned, and expressed the genes encoding for *Cp*IscS, *Cp*IscU, and *Cp*Frataxin and generated specific antibodies against these proteins. Subsequently, using western blotting and indirect immunofluorescence assays, we demonstrated that during the extracellular life cycle stages (sporozoites/merozoites), these proteins are strongly expressed (western blots; Fig. 1), while there is much less (immunofluorescent assays; Fig. 2, Figs S3–S6) or no expression (western blots; Fig. 1) in the intracellular stages of invasion. This could be due to the fact that there are relatively more host proteins in the extracts than the *C. parvum* samples. Even the previously published *Cp*Cpn60 antibody was not able to detect any protein in host cells infected with *C. parvum*. The lack of detection in western blots was not comparable to the immunofluorescent assays experiments, where all the ISC proteins were localized in most life cycle stages. This could be due to the metabolic capacity of the parasite during the different stages of its life cycle. *Cryptosporidium* is typically more metabolically active during its intracellular/epicellular stage, where there is constant exchange of nutrients with the host. This is consistent with previous transcriptomic studies, which have demonstrated that enzymes involved in the ubiquinone biosynthesis are more highly expressed at later stages of the in vitro infection [36–72 h] (Mauzy et al. 2012). The higher expression of these enzymes, which require Fe‐S clusters, could explain the need for the ISC machinery and its presence during these life cycle stages.

Using higher resolution confocal microscopy, we determined the localization of these ISC proteins in the mitosome of the sporozoites as well. Previous studies speculated that ISC machinery might be the only biosynthetic pathway present in all mitochondria (Lillig and Lill 2009). In the case of *Cryptosporidium*, indirect evidence has suggested that *Cp*IscS and *Cp*IscU are localized in their mitosomes (LaGier et al. 2003). All three generated antibodies have shown punctuate localizations of *Cp*IscS, *Cp*IscU, and *Cp*Frataxin in a structure that is no larger than 500 nm in diameter, consistent with the identified size of the mitosome in *C. parvum* sporozoite (Slapeta and Keithly 2004). This is the first direct localization of components of a biosynthetic pathway in the mitosome of *C. parvum*. It is also the first documentation of the presence of a mitosome in other life cycle stages than the sporozoite in *Cryptosporidium*. Using electron microscopy on *C. parvum‐*infected COLO‐680N cells, we have shown the presence of mitosomes in some of these intracellular/extracytoplasmic life cycle stages (Fig. S8). Interestingly, the immunofluorescent assays experiments were inconsistent in the localization pattern of the ISC proteins during the various life cycle stages in *C. parvum* infection (either punctuate or cytosolic). There are two potential explanations for this phenomenon: either the ISC machinery is translocated to the cytosol in various life cycle stages of *Cryptosporidium* or there is a morphological change of *Cryptosporidium* mitosomes in later‐stage parasites. The latter scenario might be more plausible, since it is not unusual for apicomplexan parasites. For example, the morphology of *Toxoplasma* mitochondria changes radically during the transition of the parasite from the host cell to the extracellular matrix (Ovciarikova et al. 2017). The mitochondria of *Plasmodium falciparum* grow from single, small, discrete organelles into highly branched structures in the later stages of its life cycle (van Dooren et al. 2005). Our current knowledge on the morphology of *Cryptosporidium* remnant mitochondria during the life cycle of the parasite remains limited and is worthy of further investigations. For example, the recently developed CRISPR/Cas9 system for *C. parvum* (Vinayak et al. 2015) could enable us to monitor fluorescent‐tagged single‐cells to investigate further the cellular geography of the parasite through its life cycle. Here, we have developed *Cryptosporidium‐*specific mitochondrial markers for broaden exploration of the mitosomal function and morphology.

Although the primary sequences of *Cp*IscS, *Cp*IscU, and *Cp*Frataxin proteins have conserved sequence features associated with canonical ISC functions, we also investigated whether these functions are preserved, compared with other organisms. We have examined whether *Cp*IscS could functionally replace the *S. cerevisiae* homolog in a knockout strain. Nfs1 knockout is lethal for the organism (Li et al. 1999), and thus, we have demonstrated that the *C. parvum* homolog can functionally replace the yeast counterpart, suggesting that the proteins have conserved functions.

In summary, our results have exhibited the localization of the ISC biosynthetic pathway in *C. parvum* mitosome, the first of its kind for this parasite, which will initiate new investigations on the characterization of the minimalistic organelle in *Cryptosporidium* species and could be potentially used as a model for exploring the cell biology of mitochondria of other organisms within gregarines. The ISC machinery is present in *C. parvum* mitochondria to support apoproteins that are potentially part of the ubiquinone biosynthesis. In the case of *C. andersoni*, the ISC machinery could also support the apoproteins that are present in the respiratory complex, which has been predicted to be present as well (Liu et al. 2016). The presence of both Isa1/Isa2 proteins in the predicted proteome of *C. andersoni* (Table S1)*,* which are responsible for the delivery of [4Fe‐4S] clusters to the respiratory complex, provides further support for the presence of this complex in their mitochondria. On the other hand, it is unclear why *C. ubiquitum* has to contain the ISC machinery in its mitochondria, since none of the apoproteins that require Fe‐S clusters (including ubiquinone biosynthesis) are encoded by its genome (Liu et al. 2016). To our knowledge, there are no electron micrographs revealing the *C. ubiquitum* organelle, which would be worthy of further investigations, in our quest to explore the minimal functions of mitochondrial organelles.

## Supporting information


**Figure S1.** Conservation of functionally important residues in *Cryptosporidium* cysteine desulphurase (IscS) sequences.
**Figure S2.** Conservation of functionally important residues in *Cryptosporidium* scaffold protein (IscU) sequences.
**Figure S3.** Identification of candidate functionally important residues in *Cryptosporidium* Frataxin sequences.
**Figure S4.** Detection of CpIscS via indirect immunofluorescence.
**Figure S5.** Detection of CpIscU via indirect immunofluorescence.
**Figure S6.** Detection of CpFrataxin via indirect immunofluorescence.
**Figure S7.** Work flow for the yeast‐based functional complementation assay.
**Figure S8.** Electron Microscope Images of *Cryptosporidium parvum* life cycle stages in an infected COLO‐680N culture.

**Table S1.** Identified proteins potentially involved in mitochondrial Fe‐S cluster assembly in various *Cryptosporidium* species as extracted from NCBI and CryptoDB using BLASTP.
**Table S2.** In silico mitochondrial prediction of proteins involved in Fe‐S cluster assembly in *Cryptosporidium* species.

**Video S1.** Animation of cellular staining of *Cryptosporidium parvum* cell using confocal microscopy.

## References

[jeu12663-bib-0001] Abrahamsen, M. S. , Templeton, T. J. , Enomoto, S. , Abrahante, J. E. , Zhu, G. , Lancto, C. A. , Deng, M. , Liu, C. , Widmer, G. , Tzipori, S. , Buck, G. A. , Xu, P. , Bankier, A. T. , Dear, P. H. , Konfortov, B. A. , Spriggs, H. F. , Iyer, L. , Anantharaman, V. , Aravind, L. & Kapur, V. 2004 Complete genome sequence of the apicomplexan, *Cryptosporidium parvum* . Science, 304:441–445. 15044751 10.1126/science.1094786

[jeu12663-bib-0002] Ali, V. & Nozaki, T. 2013 Iron‐sulphur clusters, their biosynthesis, and biological functions in protozoan parasites. Adv. Parasitol., 83:1–92. 23876871 10.1016/B978-0-12-407705-8.00001-X

[jeu12663-bib-0003] Ali, V. , Shigeta, Y. , Tokumoto, U. , Takahashi, Y. & Nozaki, T. 2004 An intestinal parasitic protist, *Entamoeba histolytica*, possesses a non‐redundant nitrogen fixation‐like system for iron‐sulfur cluster assembly under anaerobic conditions. J. Biol. Chem., 279:16863–16874. 14757765 10.1074/jbc.M313314200

[jeu12663-bib-0004] Basu, S. , Horakova, E. & Lukes, J. 2016 Iron‐associated biology of *Trypanosoma brucei* . Biochem. Biophys. Acta., 1860:363–370. 26523873 10.1016/j.bbagen.2015.10.027

[jeu12663-bib-0005] Boratyn, G. M. , Schaffer, A. A. , Agarwala, R. , Altschul, S. F. , Lipman, D. J. & Madden, T. L. 2012 Domain enhanced lookup time accelerated BLAST. Biol. Direct, 7:12‐6150‐7‐12. 22510480 10.1186/1745-6150-7-12 PMC3438057

[jeu12663-bib-0006] Braymer, J. J. & Lill, R. 2017 Iron‐sulfur cluster biogenesis and trafficking in mitochondria. J. Biol. Chem., 292:12754–12763. 28615445 10.1074/jbc.R117.787101 PMC5546016

[jeu12663-bib-0007] Charan, M. , Choudhary, H. H. , Singh, N. , Sadik, M. , Siddiqi, M. I. , Mishra, S. & Habib, S. 2017 Fe‐S cluster assembly in the apicoplast and its indispensability in mosquito stages of the malaria parasite. FEBS J., 284:2629–2648. 28695709 10.1111/febs.14159

[jeu12663-bib-0008] Charan, M. , Singh, N. , Kumar, B. , Srivastava, K. , Siddiqi, M. I. & Habib, S. 2014 Sulfur mobilization for Fe‐S cluster assembly by the essential SUF pathway in the *Plasmodium falciparum* apicoplast and its inhibition. Antimicrob. Agents Chemother., 58:3389–3398. 24709262 10.1128/AAC.02711-13 PMC4068488

[jeu12663-bib-0009] Claros, M. G. 1995 MitoProt, a Macintosh application for studying mitochondrial proteins. Comput. Appl. Biosci., 11:441–447. 8521054 10.1093/bioinformatics/11.4.441

[jeu12663-bib-0010] Curran, B. P. & Bugeja, V. 2014 Basic investigations in *Saccharomyces cerevisiae* . Methods Mol. Biol., 1163:1–14. 24841295 10.1007/978-1-4939-0799-1_1

[jeu12663-bib-0011] Dellibovi‐Ragheb, T. A. , Gisselberg, J. E. & Prigge, S. T. 2013 Parasites FeS up: iron‐sulfur cluster biogenesis in eukaryotic pathogens. PLoS Pathog., 9:e1003227. 23592980 10.1371/journal.ppat.1003227 PMC3617024

[jeu12663-bib-0012] van Dooren, G. G. , Marti, M. , Tonkin, C. J. , Stimmler, L. M. , Cowman, A. F. & McFadden, G. I. 2005 Development of the endoplasmic reticulum, mitochondrion and apicoplast during the asexual life cycle of *Plasmodium falciparum* . Mol. Microbiol., 57:405–419. 15978074 10.1111/j.1365-2958.2005.04699.x

[jeu12663-bib-0013] Edgar, R. C. 2004 MUSCLE: a multiple sequence alignment method with reduced time and space complexity. BMC Bioinformatics, 5:113‐2105‐5‐113. 15318951 10.1186/1471-2105-5-113 PMC517706

[jeu12663-bib-0014] Emanuelsson, O. , Brunak, S. , von Heijne, G. & Nielsen, H. 2007 Locating proteins in the cell using TargetP, SignalP and related tools. Nat. Protoc., 2:953–971. 17446895 10.1038/nprot.2007.131

[jeu12663-bib-0015] Freibert, S. A. , Goldberg, A. V. , Hacker, C. , Molik, S. , Dean, P. , Williams, T. A. , Nakjang, S. , Long, S. , Sendra, K. , Bill, E. , Heinz, E. , Hirt, R. P. , Lucocq, J. M. , Embley, T. M. & Lill, R. 2017 Evolutionary conservation and in vitro reconstitution of microsporidian iron‐sulfur cluster biosynthesis. Nat. Commun., 8:13932. 28051091 10.1038/ncomms13932 PMC5216125

[jeu12663-bib-0016] Fukasawa, Y. , Tsuji, J. , Fu, S. C. , Tomii, K. , Horton, P. & Imai, K. 2015 MitoFates: improved prediction of mitochondrial targeting sequences and their cleavage sites. Mol. Cell. Proteomics, 14:1113–1126. 25670805 10.1074/mcp.M114.043083 PMC4390256

[jeu12663-bib-0017] Gietz, R. D. & Woods, R. A. 2002 Transformation of yeast by lithium acetate/single‐stranded carrier DNA/Polyethylene glycol method. Methods Enzymol., 350:87–96. 12073338 10.1016/s0076-6879(02)50957-5

[jeu12663-bib-0018] van der Giezen, M. , Cox, S. & Tovar, J. 2004 The Iron‐sulfur cluster assembly genes iscS and iscU of *Entamoeba histolytica* were acquired by horizontal gene transfer. BMC Evol. Biol., 4:7‐2148‐4‐7. 15040816 10.1186/1471-2148-4-7 PMC373444

[jeu12663-bib-0019] Gisselberg, J. E. , Dellibovi‐Ragheb, T. A. , Matthews, K. A. , Bosch, G. & Prigge, S. T. 2013 The suf iron‐sulfur cluster synthesis pathway is required for apicoplast maintenance in malaria parasites. PLoS Pathog., 9:e1003655. 24086138 10.1371/journal.ppat.1003655 PMC3784473

[jeu12663-bib-0020] Goldberg, A. V. , Molik, S. , Tsaousis, A. D. , Neumann, K. , Kuhnke, G. , Delbac, F. , Vivares, C. P. , Hirt, R. P. , Lill, R. & Embley, T. M. 2008 Localization and functionality of microsporidian iron‐sulphur cluster assembly proteins. Nature, 452:624–628. 18311129 10.1038/nature06606

[jeu12663-bib-0021] Haussig, J. M. , Matuschewski, K. & Kooij, T. W. 2014 Identification of vital and dispensable sulfur utilization factors in the *Plasmodium* apicoplast. PLoS ONE, 9:e89718. 24586983 10.1371/journal.pone.0089718 PMC3931816

[jeu12663-bib-0022] Heiges, M. , Wang, H. , Robinson, E. , Aurrecoechea, C. , Gao, X. , Kaluskar, N. , Rhodes, P. , Wang, S. , He, C. Z. , Su, Y. , Miller, J. , Kraemer, E. & Kissinger, J. C. 2006 CryptoDB: a *Cryptosporidium* bioinformatics resource update. Nucleic Acids Res., 34:D419–D422. 16381902 10.1093/nar/gkj078 PMC1347441

[jeu12663-bib-0023] Huxley, C. , Green, E. D. & Dunham, I. 1990 Rapid assessment of *S. Cerevisiae* mating type by PCR. Trends Genet., 6:236. 2238077 10.1016/0168-9525(90)90190-h

[jeu12663-bib-0024] Karnkowska, A. , Vacek, V. , Zubacova, Z. , Treitli, S. C. , Petrzelkova, R. , Eme, L. , Novak, L. , Žárský, V. , Barlow, L. D. , Herman, E. K. , Soukal, P. , Hroudová, M. , Doležal, P. , Hroudová, M. , Stairs, C. W. , Roger, A. J. & Hampl, V. 2016 A eukaryote without a mitochondrial organelle. Curr. Biol., 26:1274–1284. 27185558 10.1016/j.cub.2016.03.053

[jeu12663-bib-0025] LaGier, M. J. , Tachezy, J. , Stejskal, F. , Kutisova, K. & Keithly, J. S. 2003 Mitochondrial‐type iron‐sulfur cluster biosynthesis genes (IscS and IscU) in the apicomplexan *Cryptosporidium parvum* . Microbiology, 149:3519–3530. 14663084 10.1099/mic.0.26365-0

[jeu12663-bib-0026] Leger, M. M. , Eme, L. , Hug, L. A. & Roger, A. J. 2016 Novel hydrogenosomes in the microaerophilic jakobid *Stygiella incarcerata* . Mol. Biol. Evol., 33:2318–2336. 27280585 10.1093/molbev/msw103 PMC4989108

[jeu12663-bib-0027] Li, J. , Kogan, M. , Knight, S. A. , Pain, D. & Dancis, A. 1999 Yeast mitochondrial protein, Nfs1p, coordinately regulates iron‐sulfur cluster proteins, cellular iron uptake, and iron distribution. J. Biol. Chem., 274:33025–33034. 10551871 10.1074/jbc.274.46.33025

[jeu12663-bib-0028] Lill, R. , Hoffmann, B. , Molik, S. , Pierik, A. J. , Rietzschel, N. , Stehling, O. , Uzarska, M. A. , Webert, H. , Wilbrecht, C. & Muhlenhoff, U. 2012 The role of mitochondria in cellular iron‐sulfur protein biogenesis and iron metabolism. Biochem. Biophys. Acta., 1823:1491–1508. 22609301 10.1016/j.bbamcr.2012.05.009

[jeu12663-bib-0029] Lillig, C. H. & Lill, R. 2009 Lights on iron‐sulfur clusters. Chem. Biol., 16:1213–1214. 20064428 10.1016/j.chembiol.2009.12.005

[jeu12663-bib-0030] Liu, S. , Roellig, D. M. , Guo, Y. , Li, N. , Frace, M. A. , Tang, K. , Zhang, L. , Feng, Y. & Xiao, L. 2016 Evolution of mitosome metabolism and invasion‐related proteins in *Cryptosporidium* . BMC Genom., 17:1006‐016‐3343‐5. 10.1186/s12864-016-3343-5 PMC5146892 27931183

[jeu12663-bib-0031] Mauzy, M. J. , Enomoto, S. , Lancto, C. A. , Abrahamsen, M. S. & Rutherford, M. S. 2012 The *Cryptosporidium parvum* transcriptome during in vitro development. PLoS ONE, 7:e31715. 22438867 10.1371/journal.pone.0031715 PMC3305300

[jeu12663-bib-0032] McGuffin, L. J. , Bryson, K. & Jones, D. T. 2000 The PSIPRED protein structure prediction server. Bioinformatics, 16:404–405. 10869041 10.1093/bioinformatics/16.4.404

[jeu12663-bib-0033] Miller, C. N. , Jossé, L. , Brown, I. , Blakeman, B. , Povey, J. , Yiangou, L. , Price, M. , Cinatl, J. , Xue, W. F. , Michaelis, M. & Tsaousis, A. D. 2018 A cell culture platform for *Cryptosporidium* that enables long‐term cultivation and new tools for the systematic investigation of its biology. Int. J. Parasitol., 48:197–201. 29195082 10.1016/j.ijpara.2017.10.001 PMC5854368

[jeu12663-bib-0034] Miller 3rd, C. A. , Martinat, M. A. & Hyman, L. E. 1998 Assessment of aryl hydrocarbon receptor complex interactions using pBEVY plasmids: expressionvectors with bi‐directional promoters for use in *Saccharomyces cerevisiae* . Nucleic Acids Res., 26:3577–3583. 9671822 10.1093/nar/26.15.3577 PMC147745

[jeu12663-bib-0035] Mogi, T. & Kita, K. 2010 Diversity in mitochondrial metabolic pathways in parasitic protists *Plasmodium* and *Cryptosporidium* . Parasitol. Int., 59:305–312. 20433942 10.1016/j.parint.2010.04.005

[jeu12663-bib-0036] Nyvltova, E. , Sutak, R. , Harant, K. , Sedinova, M. , Hrdy, I. , Paces, J. , Vlcek, C. & Tachezy, J. 2013 NIF‐type iron‐sulfur cluster assembly system is duplicated and distributed in the mitochondria and cytosol of *Mastigamoeba balamuthi* . Proc. Natl Acad. Sci. USA, 110:7371–7376. 23589868 10.1073/pnas.1219590110 PMC3645556

[jeu12663-bib-0037] Ovciarikova, J. , Lemgruber, L. , Stilger, K. L. , Sullivan, W. J. & Sheiner, L. 2017 Mitochondrial behaviour throughout the lytic cycle of *Toxoplasma gondii* . Sci. Rep., 7:42746. 28202940 10.1038/srep42746 PMC5311943

[jeu12663-bib-0038] Riordan, C. E. , Ault, J. G. , Langreth, S. G. & Keithly, J. S. 2003 *Cryptosporidium parvum* Cpn60 targets a relict organelle. Curr. Genet., 44:138–147. 12928750 10.1007/s00294-003-0432-1

[jeu12663-bib-0039] Rouault, T. A. & Maio, N. 2017 Biogenesis and functions of mammalian iron‐sulfur proteins in the regulation of iron homeostasis and pivotal metabolic pathways. J. Biol. Chem., 292:12744–12753. 28615439 10.1074/jbc.R117.789537 PMC5546015

[jeu12663-bib-0040] Ryan, U. , Paparini, A. , Monis, P. & Hijjawi, N. 2016 It's official – *Cryptosporidium* is a gregarine: what are the implications for the water industry? Water Res., 105:305–313. 27639055 10.1016/j.watres.2016.09.013

[jeu12663-bib-0041] Seeber, F. 2002 Biogenesis of iron‐sulphur clusters in amitochondriate and apicomplexan protists. Int. J. Parasitol., 32:1207–1217. 12204220 10.1016/s0020-7519(02)00022-x

[jeu12663-bib-0042] Slapeta, J. & Keithly, J. S. 2004 *Cryptosporidium parvum* mitochondrial‐type HSP70 targets homologous and heterologous mitochondria. Eukaryot. Cell, 3:483–494. 15075277 10.1128/EC.3.2.483-494.2004 PMC387664

[jeu12663-bib-0043] Small, I. , Peeters, N. , Legeai, F. & Lurin, C. 2004 Predotar: a tool for rapidly screening proteomes for N‐terminal targeting sequences. Proteomics, 4:1581–1590. 15174128 10.1002/pmic.200300776

[jeu12663-bib-0044] Stairs, C. W. , Eme, L. , Brown, M. W. , Mutsaers, C. , Susko, E. , Dellaire, G. , Soanes, D. M. , van der Giezen, M. & Roger, A. J. 2014 A SUF Fe‐S cluster biogenesis system in the mitochondrion‐related organelles of the anaerobic protist *Pygsuia* . Curr. Biol., 24:1176–1186. 24856215 10.1016/j.cub.2014.04.033

[jeu12663-bib-0045] Tsaousis, A. D. , Gentekaki, E. , Eme, L. , Gaston, D. & Roger, A. J. 2014 Evolution of the cytosolic iron‐sulfur cluster assembly machinery in *Blastocystis* species and other microbial eukaryotes. Eukaryot. Cell, 13:143–153. 24243793 10.1128/EC.00158-13 PMC3910952

[jeu12663-bib-0046] Tsaousis, A. D. , Ollagnier de Choudens, S. , Gentekaki, E. , Long, S. , Gaston, D. , Stechmann, A. , Vinella, D. , Py, B. , Fontecave, M. , Barras, F. , Lukeš, J. & Roger, A. J. 2012 Evolution of Fe/S cluster biogenesis in the anaerobic parasite *Blastocystis* . Proc. Natl Acad. Sci. USA, 109:10426–10431. 22699510 10.1073/pnas.1116067109 PMC3387072

[jeu12663-bib-0047] Tusnady, G. E. & Simon, I. 2001 The HMMTOP transmembrane topology prediction server. Bioinformatics, 17:849–850. 11590105 10.1093/bioinformatics/17.9.849

[jeu12663-bib-0048] Vinayak, S. , Pawlowic, M. C. , Sateriale, A. , Brooks, C. F. , Studstill, C. J. , Bar‐Peled, Y. , Cipriano, M. J. & Striepen, B. 2015 Genetic modification of the diarrhoeal pathogen *Cryptosporidium parvum* . Nature, 523:477–480. 26176919 10.1038/nature14651 PMC4640681

[jeu12663-bib-0049] Xu, P. , Widmer, G. , Wang, Y. , Ozaki, L. S. , Alves, J. M. , Serrano, M. G. , Puiu, D. , Manque, P. , Akiyoshi, D. , Mackey, A. J. , Pearson, W. R. , Dear, P. H. , Bankier, A. T. , Peterson, D. L. , Abrahamsen, M. S. , Kapur, V. , Tzipori, S. & Buck, G. A. 2004 The genome of *Cryptosporidium hominis* . Nature, 431:1107–1112. 15510150 10.1038/nature02977

