# Supplementary material for: Localization of Fe‐S Biosynthesis Machinery in Cryptosporidium parvum Mitosome
Source: J Eukaryot Microbiol. 2018 Jul 12;65(6):913–22. doi: 10.1111/jeu.12663 (PMC6282951; doi:10.1111/jeu.12663)
Supplement: Supplementary file 1 — Figure S1. Conservation of functionally important residues in Cryptosporidium cysteine desulphurase (IscS) sequences. Figure S2. Conservation of functionally important residues in Cryptosporidium scaffold protein (IscU) sequences. Figure S3. Identification of candidate functionally important residues in Cryptosporidium Frataxin sequences. Figure S4. Detection of CpIscS via indirect immunofluorescence. Figure S5. Detection of CpIscU via indirect immunofluorescence. Figure S6. Detection of CpFrataxin via indirect immunofluorescence. Figure S7. Work flow for the yeast‐based functional complementation assay. Figure S8. Electron Microscope Images of Cryptosporidium parvum life cycle stages in an infected COLO‐680N culture. [file JEU-65-913-s001.pdf]

SUPPORTING INFORMATION:

Localization of Fe-S Biosynthesis Machinery in *Cryptosporidium parvum* Mitosome by Christopher N. Miller, Lyne Jossé & Anastasios D. Tsaousis

Figure S1: Conservation of functionally important residues in *Cryptosporidium* cysteine desulphurase (IscS) sequences.

Figure S2: Conservation of functionally important residues in *Cryptosporidium* scaffold protein (IscU) sequences.

Figure S3: Identification of candidate functionally important residues in *Cryptosporidium* Frataxin sequences.

Figure S4: Detection of CpIscS via indirect immunofluorescence.

Figure S5: Detection of CpIscU via indirect immunofluorescence.

Figure S6: Detection of CpFrataxin via indirect immunofluorescence.

Figure S7: Work flow for the yeast-based functional complementation assay.

Figure S8: Electron Microscope Images of *C. parvum* life cycle stages in an infected COLO-680N culture.

Table S1: Identified proteins potentially involved in mitochondrial Fe-S cluster assembly in various *Cryptosporidium* species as extracted from NCBI and CryptoDB using BLASTP.

Table S2: In silico mitochondrial prediction of proteins involved in Fe-S cluster assembly in *Cryptosporidium* species.

Video S1: Animation of cellular staining of *Cryptosporidium* parvum cell using confocal microscopy

Supplementary Figure S1

```
C.parvum         -----------MIVHRYCRQWAPSVVRGISKLAFFSSMSSIAKK------------------------------------------------------RPAYFDYQATTPVDPRVLDKMM
C.hominis_UdeA01 -----------MIVHRYCRQWAPSVVRGISKVAFFSSMSSIAKK------------------------------------------------------RPAYFDYQATTPVDPRVLDKMM
C.hominis_TU502  -----------MIVHRYCRQWAPSVVRGISKVAFFSSMSSIAKK------------------------------------------------------RPAYFDYQATTPVDPRVLDKMM
C.ubiquitum      -----------MIVQGYYRQLIPGVMRSVAKVAFFSSMTSITKK------------------------------------------------------RPVYFDYQATTPVDPRVLDKMM
C.andersoni      ---------------MNMRTLPLGVSFISRHSKYLRSFSTKLKNQ--------------------------------------------------SIQRPLYFDYQATTPVDPRVLDKML
C.muris          ---------------MNMKTLPLGVSFISRRSKYLRSFSSKLKRQ--------------------------------------------------SIQRPLYFDYQATTPVDPRVLDKML
S.cerevisiae     -MLKSTATRSITRLSQVYNVPAATYRACLVSRRFYSPPAAGVKLDDNFSLETHTDIQAAAKAQASARASASGTTPDAVVASGSTAMSHAYQENTGFGTRPIYLDMQATTPTDPRVLDTML
H.sapiens        MLLRAAWRRAAVAVTAAPGPKPAAPTRGLRL-------------------------RVGDRAPQSAVPADTAAAPEV-----------------GPVLRPLYMDVQATTPLDPRVLDAML
E.coli           -----------------------------------------MKL-------------------------------------------------------PIYLDYSATTPVDPRVAEKMM

C.parvum         PFFT--EKFGNSHSRTHGYGWEAEEAVENARTNIANLIKCLPKEIIFTSGATESNNTIIRGVCDIYGDIENKKNHIITTQIEHKCVLSTLRELELKGFRVTYLKVNNKGLISLEELEKSI
C.hominis_UdeA01 PFFT--EKFGNSHSRTHGYGWEAEEAVENARTNIANLIKCLPKEIIFTSGATESNNTIIRGVCDIYGDIENKKNHIITTQIEHKCVLSTLRELELKGFRVTYLKVNNKGLISLEELEKSI
C.hominis_TU502  PFFT--EKFGNSHSRTHGYGWEAEEAVENARTNIANLIKCLPKEIIFTSGATESNNTIIRGVCDIYGDIENKKNHIITTQIEHKCVLSTLRELELKGFRVTYLKVNNKGLISLEELEKSI
C.ubiquitum      PFFT--EKFGNSHSRTHGYGWEAEDAVESARTNIANLIKCQPKEIIFTSGATESNNTIIRGVCDVYGDIKSKKNHIITTQIEHKCVLSTLRELELKGFRVTYLKVNNKGLISLEELEKSI
C.andersoni      PLYT--ELYGNSHSRTHGYGWETEDLIENARDEVAKLINCLSKEVIFTSGATESNNTIIRGVCENYGNIKEGRNHIITTQIEHKCVLSTLRELELKGFKVTYLKVDRDGLLSLKSLESAI
C.muris          PLYT--ELYGNSHSRTHGYGWETEDLIENARDEVAKLINCLSKEVIFTSGATESNNTIIRGVCENYGNIKEGRNHIITTQIEHKCVLSTLRELELKGFKVTYLEVDRDGLLSLKDLESAI
S.cerevisiae     KFYT--GLYGNPHSNTHSYGWETNTAVENARAHVAKMINADPKEIIFTSGATESNNMVLKGVPRFY---KKTKKHIITTRTEHKCVLEAARAMMKEGFEVTFLNVDDQGLIDLKELEDAI
H.sapiens        PYLI--NYYGNPHSRTHAYGWESEAAMERARQQVASLIGADPREIIFTSGATESNNIAIKGVARFY---RSRKKHLITTQTEHKCVLDSCRSLEAEGFQVTYLPVQKSGIIDLKELEAAI
E.coli           QFMTMDGTFGNPASRSHRFGWQAEEAVDIARNQIADLVGADPREIVFTSGATESDNLAIKGAANFY---QKKGKHIITSKTEHKAVLDTCRQLEREGFEVTYLAPQRNGIIDLKELEAAM

C.parvum         IPGETILASIMHVNNEIGVIQPMNLIGEICKKYNVLFHSDVAQGLGKINIDVDKWNADFLSLSAHKVYGPKGIGAFYIRSKPRRRIKPLIFGGGQERGMRSGTMPVPLAVGFGEACKIAS
C.hominis_UdeA01 IPGETILASIMHVNNEIGVIQPMNLIGEICKKYNVLFHSDVAQGLGKINIDVDKWNADFLSLSAHKVYGPKGIGAFYIRSKPRRRIKPLIFGGGQERGMRSGTMPVPLAVGFGEACKIAS
C.hominis_TU502  IPGETILASIMHVNNEIGVIQPMNLIGEICKKYNVLFHSDVAQGLGKINIDVDKWNADFLSLSAHKVYGPKGIGAFYIRSKPRRRIKPLIFGGGQERGMRSGTMPVPLAVGFGEACKIAS
C.ubiquitum      IPGETILVSIMHVNNEIGVIQPMNLIGELCKKHNVLFHSDVAQGLGKINIDVDKWNADFLSLSAHKVYGPKGIGAFYIRSKPRRRIKPLIFGGGQERGMRSGTIPVPLAVGFGEACKIAS
C.andersoni      ESGKTILVSVMFVNNEIGVIQPMESIGEVCRKHGVLFHSDVAQGLGKLQIDVNKWKVDFLSMSGHKIYGPKGIGAFYIRSKPRRRIKPLIFGGGQERGLRSGTLAVPLVVGFGEACRIAN
C.muris          ESGKTILVSVMFVNNEIGVIQSMESIGEVCRKHGVLFHSDVAQGLGKLQIDVNKWKVDFLSMSGHKIYGPKGIGAFYIRSKPRRRIKPLIFGGGQERGFRSGTLAVPLVVGFGEACRIAN
S.cerevisiae     RP-DTCLVSVMAVNNEIGVIQPIKEIGAICRKNKIYFHTDAAQAYGKIHIDVNEMNIDLLSISSHKIYGPKGIGAIYVRRRPRVRLEPLLSGGGQERGLRSGTLAPPLVAGFGEAARLMK
H.sapiens        QP-DTSLVSVMTVNNEIGVKQPIAEIGRICSSRKVYFHTDAAQAVGKIPLDVNDMKIDLMSISGHKIYGPKGVGAIYIRRRPRVRVEALQSGGGQERGMRSGTVPTPLVVGLGAACEVAQ
E.coli           RD-DTILVSIMHVNNEIGVVQDIAAIGEMCRARGIIYHVDATQSVGKLPIDLSQLKVDLMSFSGHKIYGPKGIGALYVRRKPRVRIEAQMHGGGHERGMRSGTLPVHQIVGMGEAYRIAK

C.parvum         SEMNSDSIHVKSLYDKLYKGITTQLPDVELNGCGVNRMFGNLNLSFTGVEGESLMMKLYSLALSSGSACTSASLEPSYVLRAIGVGEDVAHTSIRFGLGRFTKHEDVDKAVKEIVESVTL
C.hominis_UdeA01 SEMNSDSIHVKSLYDKLYKGITTQLPDVELNGCGVNRMFGNLNLSFTGVEGESLMMKLYSLALSSGSACTSASLEPSYVLRAIGVGEDVAHTSIRFGLGRFTKHEDVDKAVKEIVESVTL
C.hominis_TU502  SEMNSDSIHVKSLYDKLYKGITTQLPDVELNGCGVNRMFGNLNLSFTGVEGESLMMKLYSLALSSGSACTSASLEPSYVLRAIGVGEDVAHTSIRFGLGRFTKHEDVDKAVKEIVESVTL
C.ubiquitum      SEMNLDSIHVKSLYDKLYKGITTQLPDVELNGCSINRMFGNLNLSFAGVEGESLMMKLYSMALSSGSACTSSSLEPSYVLRAIGVGEDVAHTSIRFGLGRFTRHEDVDKAVKEIVESVTL
C.andersoni      DEMVRDSIHVKKLYHRLYHGIQSQVPNIKLNGSPIHRCFNNLNLSFAGVEGESLLMKLQSLALSSGSACTSASLEPSYVLRAIGIDEETAHTSIRFGLGRFTTPSEIDLAVDEVVKAVSS
C.muris          DEMVRDSIHVKKLYHKLYHGIQSQVPNIKLNGSSIHRCFNNLNLSFAGVEGESLLMKLQSLALSSGSACTSASLEPSYVLRAIGVDEETAHTSIRFGLGRFTTPSEIDLAVDEVVKAVSS
S.cerevisiae     KEFDNDQAHIKRLSDKLVKGLL-SAEHTTLNGSPDHRYPGCVNVSFAYVEGESLLMALRDIALSSGSACTSASLEPSYVLHALGKDDALAHSSIRFGIGRFSTEEEVDYVVKAVSDRVKF
H.sapiens        QEMEYDHKRISKLSERLIQNIMKSLPDVVMNGDPKHHYPGCINLSFAYVEGESLLMALKDVALSSGSACTSASLEPSYVLRAIGTDEDLAHSSIRFGIGRFTTEEEVDYTVEKCIQHVKR
E.coli           EEMATEMERLRGLRNRLWNGIK-DIEEVYLNGDLEHGAPNILNVSFNYVEGESLIMALKDLAVSSGSACTSASLEPSYVLRALGLNDELAHSSIRFSLGRFTTEEEIDYTIELVRKSIGR

C.parvum         LRKMSPLWDSITDTKVKDEDSLKWT--
C.hominis_UdeA01 LRKMSPLWDSITDTKVKDEESLKWT--
C.hominis_TU502  LRKMSPLWDSITDTKVKDEESLKWT--
C.ubiquitum      LRKMSPLWDIDTGTRVKNEESLKWT--
C.andersoni      LRNMSPIWDQINSTNPSDISSPIWT--
C.muris          LRNMSPIWDQINSTNSSDISGPIWT--
S.cerevisiae     LRELSPLWEMVQEG--IDLNSIKWSGH
H.sapiens        LREMSPLWEMVQDG--IDLKSIKWTQH
E.coli           LRDLSPLWEMYKQG--VDLNSIEWAHH
```

Supplementary Figure S2

```
C.parvum          -MLQLRQLIDKRILIKKCVPICQRLFYSDTVHDHFRNPRNVGSLPSDDKNVGTAVVGKASCGDVVKLQVDIRD--GIIKDAKFKTFGCGSAIASTSYATELIIGKTTEEALKINNKTIAD
C.hominis UdeA01  -MLQLRQLIDKRILIKKCVPICQRLFYSDTVHDHFRNPRNVGSLPSDDKNVGTAVVGKASCGDVVKLQVDIRD--GIIKDAKFKTFGCGSAIASTSYATELIIGKTTEEALKINNKTIAD
C.hominis TU502   -MLQLRQLIDKRILIKKCVPICQRLFYSDTVHDHFRNPRNVGSLPSDDKNVGTAVVGKASCGDVVKLQVDIRD--GIIKDAKFKTFGCGSAIASTSYATELIIGKTTEEALKINNKTIAD
C.ubiquitum       -MLQLRNFVDKRILLKKCVPICQRLFYSNVVNDHFRNPRNVGSLPSDDKNVGTAVVGKAACGDVVKLQVSIKD--GVIKDAKFKTFGCGSAIASTSYATELIIGKTTEEALKVNNKTIAN
C.andersoni       MMIVVRSCITR--CKQHLSLIAQKAMYSEAVHDHFRNPRNVGTLPTDKKNVGTAVVGKAACGDVVKLQVSIED--GIIRDAKFKTFGCGSAIASSSYVTELIKGKTPEDALKIKNTDIAN
C.muris           MMIAVRNCITR--CKQHLSLIAQKAMYSEAVHDHFRNPRNVGTLPSDKKNVGTAVVGKAACGDVVKLQVSIED--GVIRDAKFKTFGCGSAIASSSYVTELIKGKTPEDALKIKNTDIAN
S.cerevisiae      MFARLANPAHFKPLTGSHITRAAKRLYHPKVIDHYTNPRNVGSMDKSLANVGTGIVGAPACGDVIKLQIQVNDKSGIIENVKFKTFGCGSAIASSSYMTELVRGMSLDEAVKIKNTEIAK
H.sapiens         -----------------MVLIDMSVDLSTQVVDHYENPRNVGSLDKTSKNVGTGLVGAPACGDVMKLQIQVDEK-GKIVDARFKTFGCGSAIASSSLATEWVKGKTVEEALTIKNTDIAK
E.coli            ------------------------MAYSEKVIDHYENPRNVGSFDNNDENVGSGMVGAPACGDVMKLQIKVNDE-GIIEDARFKTYGCGSAIASSSLVTEWVKGKSLDEAQAIKNTDIAE


C.parvum          HLNLPPIKLHCSLLAEDAIKHAIKNYQDKQLKS-----------------------
C.hominis UdeA01  HLNLPPIKLHCSLLAEDAIKHAIKNYQDKQLKS-----------------------
C.hominis TU502   HLNLPPIKLHCSLLAEDAIKHAIKNYQDKQLKS-----------------------
C.ubiquitum       HLNLPPIKLHCSLLAEDAIKHAIKNYQEKQQES-----------------------
C.andersoni       YLNLPPVKLHCSLLAEDAVHLAIKDYQSKQSTSDDKSSSSVNISYSDDNKVNPNTK
C.muris           YLNLPPVKLHCSLLAEDAVHLAIKDYQSKQSTSDDKSSSSVNISYSDDNKVDPDTK
S.cerevisiae      ELSLPPVKLHCSMLAEDAIKAAIKDYKTKRNPSVLH--------------------
H.sapiens         ELCLPPVKLHCS-------KSVLFPAEEKTQLSP----------------------
E.coli            ELELPPVKIHCSILAEDAIKAAIADYKSKREAK-----------------------
```

Supplementary Figure S3

```
C.parvum Iowa  ------------MNSIKLLNLKVVQNLSKFNTQYKVFNFRKSISTLKFLCDRNSKFVPPL
C.hominis      ------------MNSIKLLNLKVVQNLSKFNTPYKVFNFRKSISTLKFLCDSNPKFVPPL
C.ubiquitum    ------------MNSIKLLNLKIIQNLNRFNSSCKIFNFRNSAIPLKFLFDNNYKLYVLK
C.andersoni    ------------MKCITIFNIQII----GSLFKKSQILSKKISFKYFCNND----FISIR
C.muris        ------------MKCIAIFNIQTI----GSLFKKSQILSKKIPFKYFCNND----LISIR
S.cerevisiae   ------------MIKRSL------------ASLVRVSSVMGRRYMIAAAGGERARFCPAV
H.sapiens      MWTLGRRAVAGLLASPSPAQAQTLTRVPRPAELAPLCGRRGLRTDIDATCTPRRASSNQR
E.coli         --------------------------------------------------MND-------

C.parvum Iowa  AHN-LLKLKKNRIYTNSSDIS--------------QFSSKTEGLFFEIEKKLSDLLD--N
C.hominis      AHN-LLKLKKNRIYTNSSDIS--------------QFSSKTEGLFFEIEKKLSDLLD--N
C.ubiquitum    AHS-ILTIQKSRINTNSLDIS--------------QFNLKTETLFFEIESKLNDLLD--S
C.andersoni    KFS-I--INKYPKYFTTISIA--------------DFNNKADQLLHIISEKIINTQD--Q
C.muris        KFS-I--INKYPKYFTTTSIA--------------DFNNKADQLLHIISEKIINTQD--Q
S.cerevisiae   TNKKNHTVNTFQKRFVESSTDGQVVPQEVLNLPLEKYHEEADDYLDHLLDSLEELSEAHP
H.sapiens      GLNQIWNVKKQSVYLMNLRKSGTLGHPGSLD--ETTYERLAEETLDSLAEFFEDLAD-KP
E.coli         --------------------S--------------EFHRLADQLWLTIEERLDDWDG--D
                                                                α1

C.parvum Iowa  GLLSDIDL--HDEFMNISFNLNGEK---NTIVISKQPATNQIWYSSPLRKPDYFEF-NSD
C.hominis      GLLSDIDL--HDEFMNISFNLNGEK---NTIVISKQPATNQIWYSSPLRKPDYFEF-NSD
C.ubiquitum    GFLSDIDL--HDEFMNITFNLNGVK---NTIVISKQPATKQIWYSSPLRKPDYFEF-NSD
C.andersoni    NLIDDIDC--HDEFLNITFRYKDRQSSSGTIVISKQSATKQMWYSSPMRPPDYFEF-ENN
C.muris        NLIDDIDC--HNEFLNITFRYKDRQ-SSGTIVISKQSATKQIWYSSPIRPPDYFEF-ESN
S.cerevisiae   DCIPDVEL--SHGVMTLEIP------AFGTYVINKQPPNKQIWLASPLSGPNRFDLLDGE
H.sapiens      YTFEDYDVSFGSGVLTVKLG--GDL---GTYVINKQTPNKQIWLSSPSSGPKRYDWTGKN
E.coli         ---SDIDCEINGGVLTITFE-NGSK-----IIINRQEPLHQVWLATK-QGGYHFDLKGDE
                   β1        β2             β3           β4       β5

C.parvum Iowa  WRSNRTNKTLFEVLNDDLYKATGIHVNL----------
C.hominis      WRSNRTNKTLFEVLNDDLFKATGIHVNL----------
C.ubiquitum    WRSNRTNETLFEALHDDLFKATGIHVNF----------
C.andersoni    WKSKRSGLSLFEVLESDLFEGTGLKFNFLEK-------
C.muris        WKSKRSGLSLFEALESDLFEGTGLKFNFLEK-------
S.cerevisiae   WVSLRNGTKLTDILTEEVEKAISKSQ------------
H.sapiens      WVFSHDGVSLHELLAAELTKALKTKLDLSWLAYSGKDA
E.coli         WICDRSGETFWDLLEQAATQQAGETVSFR---------
               β6            α2
```

Supplementary Figure 4

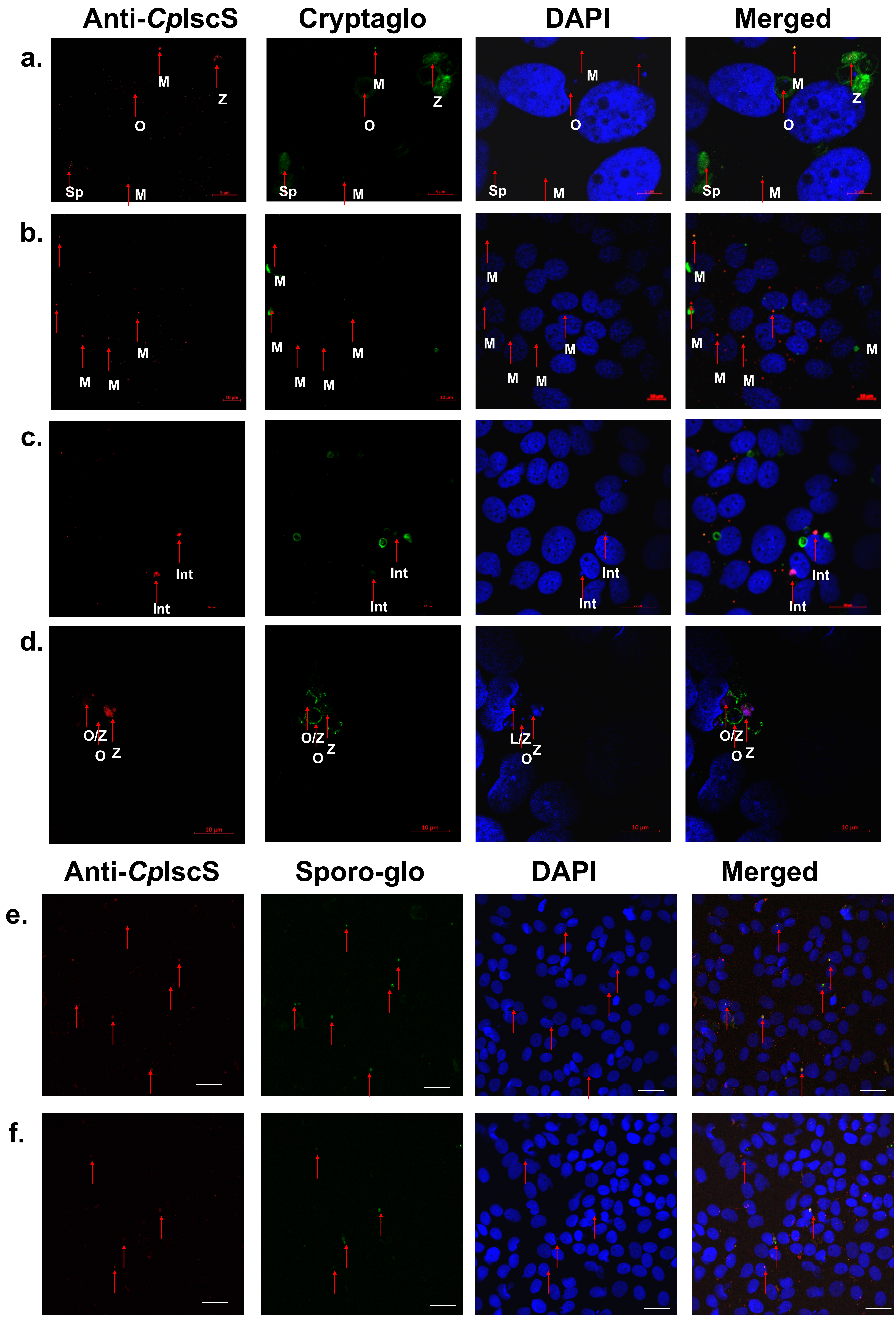

Supplementary Figure S5

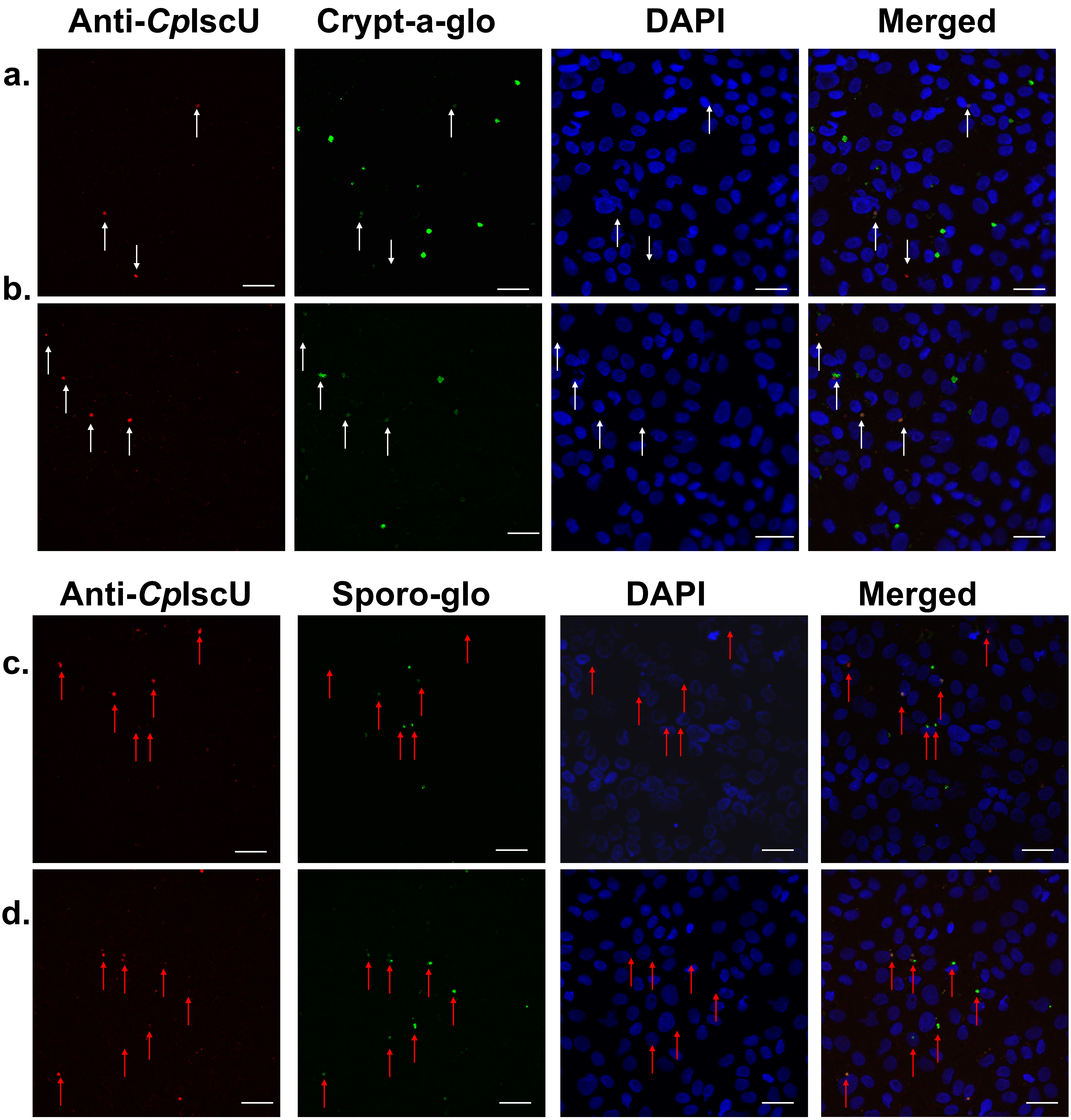

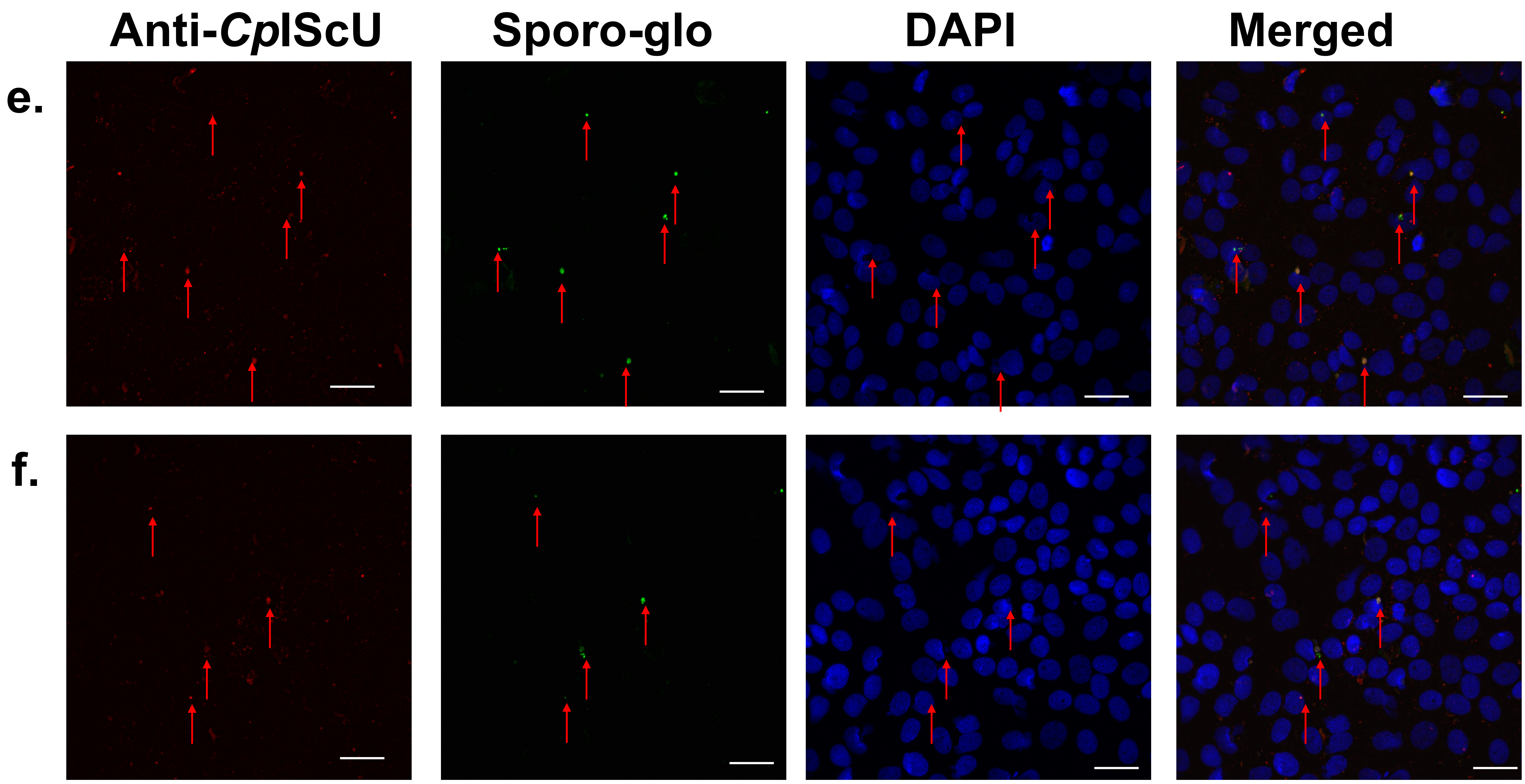
Anti-*Cp*IScU
Sporo-glo
DAPI
Merged
e.
f.

Supplementary Figure 6

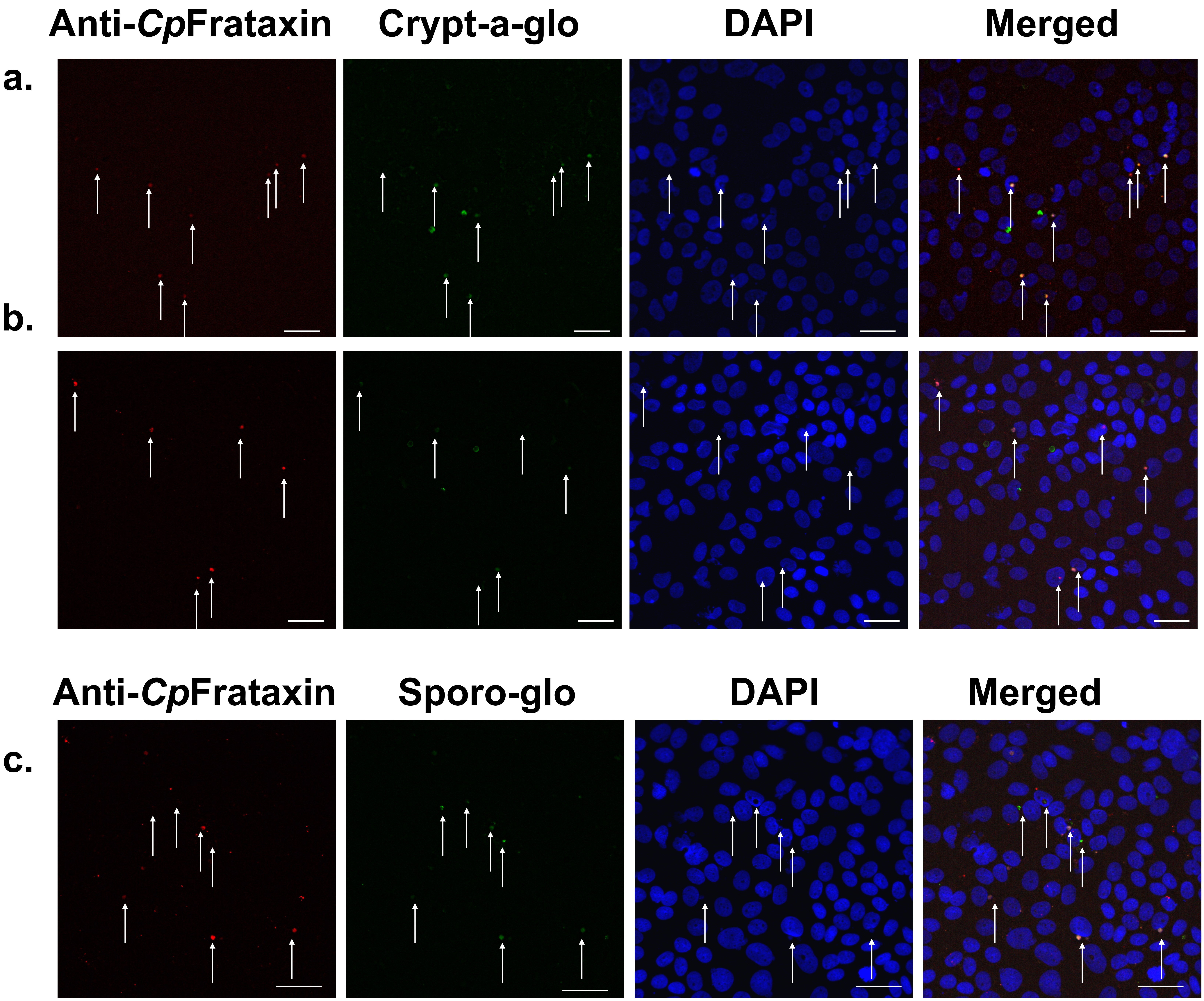

Supplementary Figure S7

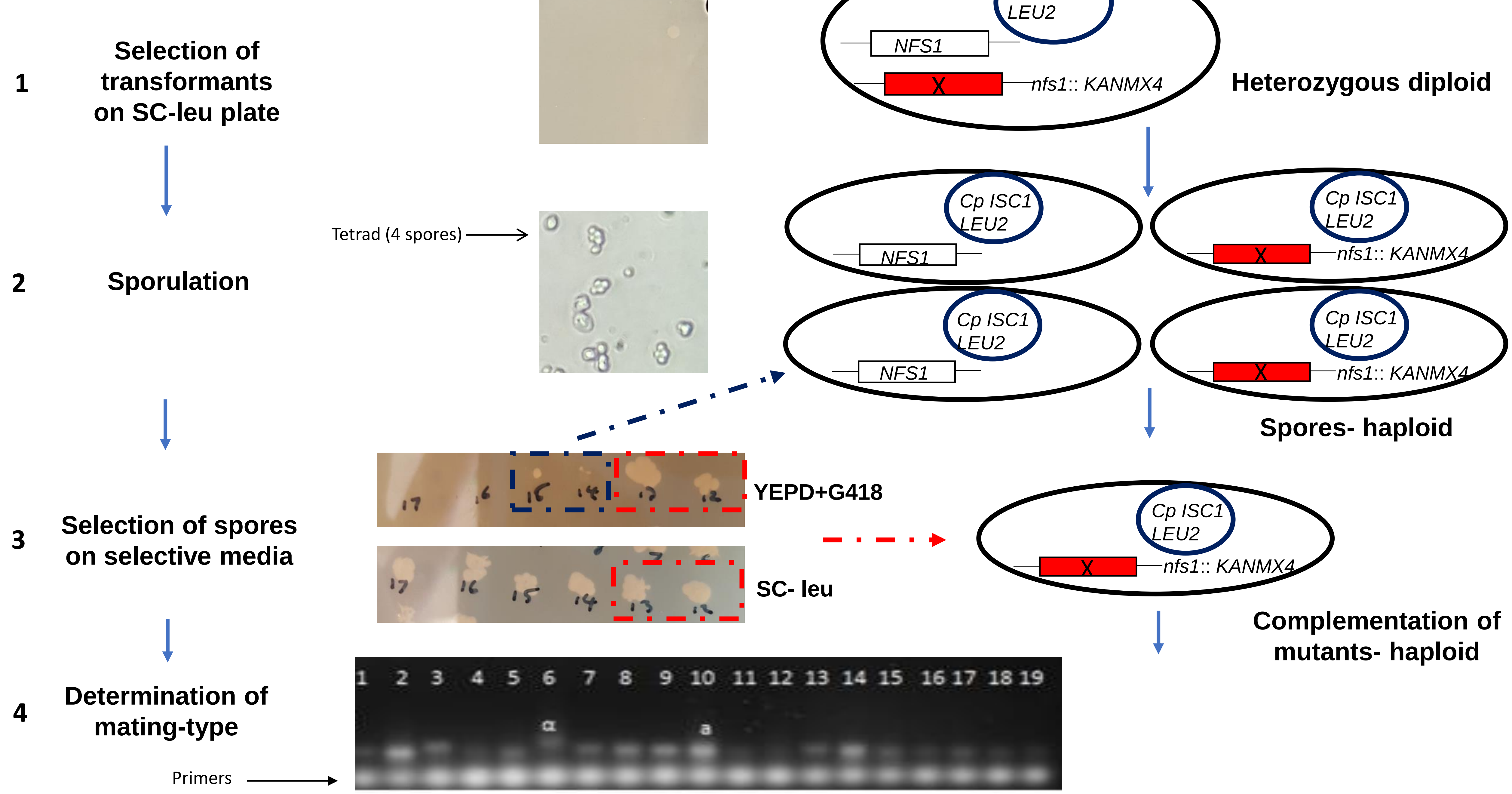

Supplementary Figure S8

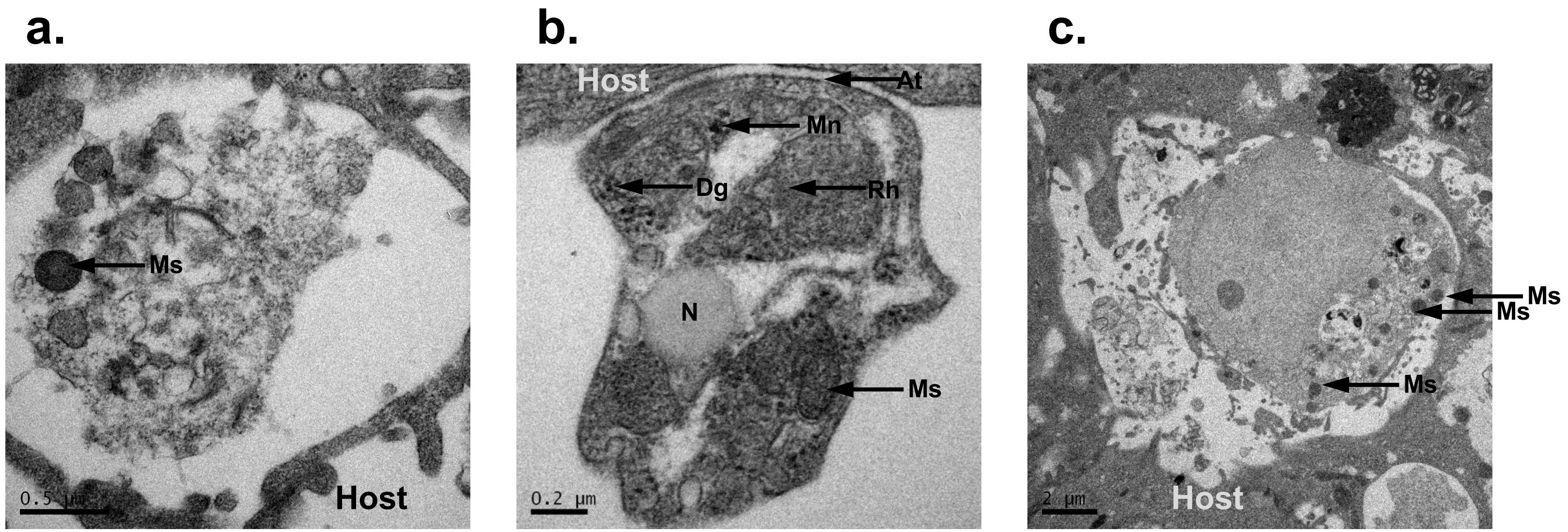

Supplementary Table 2: ***In silico* mitochondrial prediction of proteins involved in Fe-S cluster assembly in *Cryptosporidium* species**

Species	Protein	MitoFates	Predotar	MitoProt	TargetP
C. parvum	IscS	0.877	0.76	0.9930	0.849
	IscU	0.889	0.50	0.9617	0.418
	Frataxin	0.273	0.13	0.9485	0.586
	Arh1	0	0	0.1862	0.012
	Yah1	0.932	0.91	0.9941	0.946
	MFRN1/Mrs3	0.017	0.00	0.0230	0.042
	MFRN2/Mrs4	0.04	0.00	0.2602	0.016
	Grx5	0	0.00	0.0965	0.021
	mtHsp70	0.258	0.08	0.9877	0.741
	Jac1	0.247	0.00	0.2205	0.187
	Mge1	0.854	0.69	0.9674	0.887
	Ind1	0.008	0.00	0.0737	0.093
	Isa1	-	-	-	-
	Isa2	-	-	-	-
	Atm1	0.373	0.69	0.6661	0.581
	Erv1	-	-	-	-
C. hominis	IscS	0.819	0.72	0.9888	0.852
	IscU	0.889	0.50	0.9617	0.418
	Frataxin	0.243	0.16	0.8810	0.583
	Arh1	0.045	0.06	0.4090	0.148
	Yah1	-	-	-	-
	MFRN1/Mrs3	0.020	0.00	0.0230	0.041
	MFRN2/Mrs4	0.051	0.01	0.1975	0.020
	Grx5	0.004	0.00	0.0406	0.067
	mtHsp70	0.44	0.05	0.9329	0.548
	Jac1	0.009	0.00	0.0091	0.244
	Mge1	0.864	0.69	0.9695	0.866
	Ind1	0.117	0.02	0.6708	0.337
	Isa1	-	-	-	-
	Isa2	-	-	-	-
	Atm1	0.228	0.69	0.7737	0.573
	Erv1	-	-	-	-
C. ubiquitum	IscS	0.887	0.52	0.9750	0.691
	IscU	0.958	0.48	0.9303	0.582
	Frataxin	0.492	0.10	0.9706	0.671
	Arh1	0.055	0.24	0.1361	0.049
	Yah1	0.716	0.24	0.9762	0.753
	MFRN1/Mrs3	0.016	0.00	0.0472	0.039
	MFRN2/Mrs4	-	-	-	-
	Grx5	0	0.00	0.2028	0.067
	mtHsp70	0.131	0.14	0.9588	0.717

	Jac1	0.009	0.00	0.0069	0.244
	Mge1	0.622	0.23	0.9778	0.748
	Ind1	0.277	0.03	0.8964	0.569
	Isa1	-	-	-	-
	Isa2	-	-	-	-
	Atm1	0.372	0.49	0.8801	0.619
	Erv1	-	-	-	-
C. andersoni	IscS	0.953	0.58	0.9230	0.942
	IscU	0.865	0.34	0.8425	0.640
	Frataxin	0.079	0.12	0.8360	0.156
	Arh1	0.835	0.87	0.7677	0.882
	Yah1	0	0.02	0.1832	0.176
	MFRN1/Mrs3	0.010	0.00	0.0579	0.077
	MFRN2/Mrs4	-	-	-	-
	Grx5	0.022	0.13	0.2324	0.068
	mtHsp70	0.543	0.00	0.9197	0.863
	Jac1	0.006	0.61	0.0354	0.255
	Mge1	0.117	0.01	0.9886	0.462
	Ind1	0.012	0.02	0.0537	0.040
	Isa1	0.003	0.02	0.3211	0.163
	Isa2	0.580	0.47	0.8120	0.749
	Atm1	-	-	-	-
	Erv1	0.009	0.02	0.0777	0.064
C. muris	IscS	0.931	0.77	0.9843	0.952
	IscU	0.897	0.19	0.8456	0.656
	Frataxin	0.083	0.17	0.7823	0.196
	Arh1	0.792	0.90	0.8725	0.915
	Yah1	0	0.01	0.1844	0.092
	MFRN1/Mrs3	0.008	0.00	0.0565	0.064
	MFRN2/Mrs4	0.018	0.00	0.0348	0.045
	Grx5	0	0.00	0.1164	0.032
	mtHsp70	0.382	0.16	0.9464	0.798
	Jac1	0.010	0.00	0.0252	0.282
	Mge1	0.112	0.64	0.9896	0.621
	Ind1	0.019	0.01	0.0706	0.062
	Isa1	0	0.01	0.4522	0.166
	Isa2	0.976	0.63	0.8293	0.759
	Atm1	0.320	0.46	0.9362	0.683
	Erv1	0.013	0.02	0.0982	0.071

In silico prediction of potential mitochondrion-targeted proteins using MitoFates (Fukasawa et al. 2015) and displaying the "possibility of presequence", MitoProt (Claros 1995) while displaying the "probability of export to mitochondria", Predotar (Small et al. 2004) and TargetP (Emanuelsson et al. 2007), by displaying the probability of prediction of mitochondrial targeting signal. Possibilities above 85 % are shaded in light gray for MitoFates, MitoProt and Target P and above 65 % for Predotar.
